# Functional, patient-derived 3D tri-culture models of the uterine wall in a microfluidic array

**DOI:** 10.1093/humrep/deae214

**Published:** 2024-09-15

**Authors:** Caroline Busch, Christopher J Hill, Karla Paterson, Ronan Mellin, Michele Zagnoni, Dharani K Hapangama, Mairi E Sandison

**Affiliations:** Department of Biomedical Engineering, University of Strathclyde, Wolfson Centre, Glasgow, UK; Department of Electronic and Electrical Engineering, Centre for Microsystems & Photonics, University of Strathclyde, Glasgow, UK; Department of Women’s and Children’s Health, Institute of Life Course and Medical Sciences, University of Liverpool, Member of Liverpool Health Partners, Liverpool, UK; Liverpool Women’s Hospital NHS Foundation Trust, Member of Liverpool Health Partners, Liverpool, UK; ScreenIn3D Limited, Glasgow, UK; ScreenIn3D Limited, Glasgow, UK; Department of Electronic and Electrical Engineering, Centre for Microsystems & Photonics, University of Strathclyde, Glasgow, UK; ScreenIn3D Limited, Glasgow, UK; Department of Women’s and Children’s Health, Institute of Life Course and Medical Sciences, University of Liverpool, Member of Liverpool Health Partners, Liverpool, UK; Liverpool Women’s Hospital NHS Foundation Trust, Member of Liverpool Health Partners, Liverpool, UK; Department of Biomedical Engineering, University of Strathclyde, Wolfson Centre, Glasgow, UK

**Keywords:** microfluidics, 3D culture, endometrium, myometrium, decidualization, uterine wall, patient-derived

## Abstract

**STUDY QUESTION:**

Can a functional *in vitro* model, containing the main cellular components of the uterine wall, be generated from cells derived from patient tissues?

**SUMMARY ANSWER:**

We present a three-dimensional (3D) physiologically relevant, organ-on-a-chip model of the uterine wall containing primary endometrial and myometrial cellular participants, generated from human uterine tissue.

**WHAT IS KNOWN ALREADY:**

As a highly dynamic reproductive organ, the human uterus plays fundamental physiological roles in menstruation and childbirth. The endometrial–myometrial junction (EMJ) defines the interface between the inner mucosal layer (endometrium) and outer smooth muscle zone (myometrium) that comprises the uterine wall. The EMJ is implicit in several uterine pathologies of unknown aetiology, including adenomyosis and abnormally invasive placenta; however, despite this, no patient-derived *in vitro* models of the uterine wall containing all EMJ participants currently exist.

**STUDY DESIGN, SIZE, DURATION:**

We employed microfluidic technology to characterize multiple miniaturized models of the uterine wall. Protocols were tested that included variations in the seeding order of endometrial and myometrial fractions, and the addition of a low viscosity extracellular matrix to influence cell behaviour. Ultimately, functional hormone responses of patient-derived uterine wall models were assessed.

**PARTICIPANTS/MATERIALS, SETTING, METHODS:**

Endometrial (n = 9) and myometrial biopsies (n = 4) were enzymatically dissociated to create epithelial, stromal and myometrial cellular fractions. Cell suspensions were seeded into non-adhesive poly(dimethylsiloxane) microfluidic devices containing 5 × 5 microwell arrays. The fate of individual cell types was monitored in real-time using fluorescent tracers, and cell phenotype was characterized by immunocytochemistry. Model functionality was assessed by measuring Ca^2+^ responses to agonist stimulation, and both insulin-like growth factor binding protein 1 (IGFBP-1) and osteopontin secretion in response to hormone stimulation.

**MAIN RESULTS AND THE ROLE OF CHANCE:**

When subjected to microfluidic culture in isolation, endometrial stromal cells and smooth muscle myocytes formed compact spheroids, whilst epithelial cells produced diffuse aggregates. Tri-cultures were established by sequential seeding of individual or combined cell fractions at various ratios. Regardless of the protocol, epithelial cells localized to the outer periphery of tri-culture spheroids, which varied in morphology across the protocols. Incorporation of 5% [v/v] Matrigel^®^ improved the reproducibility of 3D aggregates which exhibited robust self-assembly of a stromal/smooth muscle core encased in epithelium. Exposure of tri-cultures to oestradiol, medroxyprogesterone acetate and cyclic adenosine monophosphate (cAMP) increased secretion of IGFBP-1, which indicates stromal decidualization, and enhanced epithelial cell osteopontin secretion. Stimulation with endothelin-1 induced Ca^2+^ signalling in myocytes.

**LIMITATIONS, REASONS FOR CAUTION:**

Endometrial and myometrial tissue was collected from relatively few donors. Myometrial tissue was collected from pregnant donors, which may have influenced the myocyte phenotype. Furthermore, endometrial tissue sampling was from women not having a hysterectomy, thus may not include the deeper basalis region, which may limit the physiological mimicry of the final models.

**WIDER IMPLICATIONS OF THE FINDINGS:**

Our novel approach to modelling the uterine wall in 3D captures all of the main cell types in a medium-throughput system, enabling the screening of hundreds of cultures in parallel from a single biopsy. This system shows great promise for examining the cellular interplay between physiological cues and EMJ pathologies, such as the impact of uterine peristalsis and cyclical hormones on the pathogenesis of adenomyosis.

**STUDY FUNDING/COMPETING INTEREST(S):**

C.B. was supported by an Organ-on-a-Chip Technologies Network Pump Priming Project grant. C.J.H. was supported by a Wellbeing of Women project grant (RG2137), SRI/Bayer and Wellcome Trust IFFS3. D.K.H. was supported by a Wellbeing of Women project grant (RG2137) and MRC clinical research training fellowship (MR/V007238/1). M.Z. is Director and Co-Founder of ScreenIn3D Limited. The other authors declare no conflict of interest.

**TRIAL REGISTRATION NUMBER:**

N/A.

## Introduction

The human uterus is a central organ in a woman’s reproductive system, responsible for fundamental physiological processes including menstruation, pregnancy, and childbirth. The uterus comprises an inner mucosal lining (endometrium), a smooth muscle layer (myometrium), and outer serosa. The endometrium is the site of embryo implantation and pregnancy establishment, whilst the myometrium functions both as a protective shell for the foetus before labour and provides the muscular contractions to expel the pregnancy during labour (Hertelendy and Zakar, 2004; Tempest *et al.*, 2022). The endometrium is arranged in two sub-luminal layers composed primarily of epithelial glands and stromal cells; the superficial functionalis and underlying basalis. Both the lumen and functionalis are shed during menses and are regenerated during the proliferative (follicular) and secretory (luteal) phases in response to ovarian sex hormones (Tempest *et al.*, 2022; Maclean *et al.*, 2020a).

The endometrial–myometrial junction (EMJ) is the junctional zone between the mucosal and smooth muscle zones of the uterus. The smooth muscle layer directly adjacent to the endometrium, the inner myometrium, is physiologically distinct from the outer myometrium; contractions originating from the inner myometrium regulate uterine peristalsis in a cyclical rhythm orchestrated by oestrogen and progesterone (Naftalin and Jurkovic, 2009). The EMJ is implicit in physiological preservation of basalis endometrium and in several pathologies, including adenomyosis and placenta accreta disorders (Tempest *et al.*, 2022). The endometrial basalis harbours stem/progenitor cells responsible for iterative endometrial regeneration, whilst the inner myometrium/EMJ is proposed to maintain basalis cells quiescent by being less responsive to ovarian hormonal signals (Kamal et al., 2016; Maclean *et al.*, 2020b). Adenomyosis occurs when endometrial tissue is present deep within the myometrium, likely via invagination of the endometrial basalis through the EMJ. This process may be initiated by both physical and physiological trauma to the EMJ (Kunz *et al.*, 2005; Leyendecker and Wildt, 2011; Hao *et al.*, 2020; Yamaguchi *et al.*, 2021). The placenta accreta spectrum describes a range of pathologies characterized by abnormal invasion of the placenta into the myometrium. This invasion is patently linked to processes that disrupt the EMJ, such as Caesarean section (CS) (Jansen *et al.*, 2020).

Three-dimensional (3D) *in vitro* models better recapitulate structural organization and functional cellular interactions than traditional two-dimensional (2D) cultures, and a variety of 3D uterine models exist. Most, however, incorporate endometrial cells alone, in many cases using cell lines rather than patient-derived cells (Gołąbek-Grenda and Olejnik, 2022) and were often developed as models of implantation (Rawlings *et al.*, 2021; Li *et al.*, 2022) or for studies into gynaecological cancers (Park *et al.*, 2021; Yang *et al.*, 2021). Far fewer studies have reported on the development of 3D myometrial models (Vidimar *et al*, 2018; Xie *et al*, 2018). Lü *et al* (2009) previously reported on a three-layered rabbit uterine tissue model and Kuperman *et al* (2020) have recently described a tri-culture uterine wall macro-model based on cell lines. However, despite the significance of the EMJ in uterine physiology and prevalent pathologies, no patient-derived *in vitro* models containing all key EMJ cellular participants (i.e. epithelia, stroma and myocytes) currently exist. The development of myometrial-endometrial tri-cultures from small quantities of uterine tissue are urgently required to mimic the uterine wall with a patient-specific focus, facilitating the study of physiological and disease mechanisms.

Miniaturization of *in vitro* models using microfluidic, organ-on-a-chip (OOAC) technologies offers significant opportunities (Campo *et al.*, 2020) to better mimic the *in vivo* microenvironment and to scale-up the screening of limited but highly relevant patient-derived models. Such OOAC systems have recently been utilized to generate endometrium models (Gnecco *et al.*, 2019; Campo *et al.*, 2020), including a 3D model of vascularized endometrium that incorporated human epithelial, stromal and endothelial cell lines within a fibrin hydrogel (Ahn *et al.*, 2021). More complex systems have also been established, including a physio-mimetic model of the menstrual cycle comprising a multi-chamber platform containing cells of the ovaries, oviducts, cervix and endometrium (Xiao *et al.*, 2017). However, none of these systems were used to model a 3D uterine wall by incorporating myometrial cells and, importantly, did not take advantage of the OOAC miniaturization and multiplexing capabilities, which are key to maximizing the utility of patient tissue when developing screening and functional assays using patient-derived cells.

In this study, we have validated for the first time a 3D, premenopausal, patient-derived tri-culture model of the uterine wall, sequentially incorporating endometrial epithelial and stromal cells, and myometrial cells (the three principal cell types of the EMJ). The 3D models, which were cultured for up to 13 days, were created within arrays of microwells embedded within a multi-channel microfluidic device, allowing multiple miniaturized assays to be performed in a single experiment for investigating structural and functional phenotype. Here, we present this new methodology and describe its optimization, the characterization of cellular organization, and subsequent functional assays, including hormonal stimulation to elicit a decidualization response. The 3D multicellular cultures derived from patient biopsies offer an effective route to better mimic the biological complexity of uterine tissue, and our model provides a new tool for understanding cellular behaviours in uterine pathologies. Furthermore, with substantial donor–donor heterogeneity in disease presentation and in response to treatment, our patient-derived models ultimately have potential for application in personalized screening of therapies.

## Materials and methods

### Ethical approval for the collection and use of human tissue

The collection and use of human tissue was approved by the Liverpool Adult Research Ethics Committee (LREC: 19/SC/0449) and all women recruited gave informed written consent.

### Primary cell isolation

Endometrial pipelle biopsies were collected from premenopausal women undergoing gynaecological surgery for benign conditions who were not taking hormonal medications. Myometrial biopsies were collected during pre-labour elective CS deliveries of women at full-term pregnancies (>37 weeks). An overview of participant demographics and the experiments for which their samples were used is given in Supplementary Table S1. Tissue was minced into small pieces (<1 mm) using a scalpel blade and digested with 1 mg/ml Dispase II (Gibco™, Thermo Fisher Scientific, UK), 2 mg/ml collagenase type I (Gibco™, Thermo Fisher Scientific) and 80 µg/ml deoxyribonuclease (DNase) I (Merck, UK) for ∼1 h at 37°C in a shaking water bath as previously described (Valentijn *et al.*, 2013). Digests were periodically triturated to enhance tissue breakdown and observed under a microscope to check for the presence of whole tissue fragments and free epithelial glands. The endometrial digests were passed through a 40 µm cell sieve (Falcon™) to separate glandular (retentate) and stromal (flow-through) elements. Erythrocytes were removed from the myometrial digests and endometrial stromal fractions using Ficoll-Paque (Merck) density gradient centrifugation. Intact endometrial glands, endometrial stroma and myometrial cells were frozen and stored at −80°C prior to use.

### Primary cell culture

Primary endometrial tissue samples and myometrial smooth muscle cells (SMCs) were thawed rapidly at 37°C and immediately washed in 5 ml cell culture medium (complete for SMCs and stroma, basal for epithelia). To further digest large epithelial glands, pellets were resuspended in 1 ml serum-free media (SFM; phenol red-free DMEM/F12, Merck, UK), 1 ml 0.25% trypsin (Thermo Fisher Scientific) and 100 µl DNase I (4 mg/ml, Merck) and incubated at 37°C for 20 min. Glands were gently triturated using a narrow bore pipette tip to yield single cells. Following trituration, the digestion reaction was quenched with 1 ml foetal bovine serum (FBS, Thermo Fisher Scientific). The digested epithelial fraction was washed with complete cell culture medium (phenol red-free DMEM/F12, 10% [v/v] FBS (Merck), 2 mM L-glutamine and 100 µg/ml Primocin (InvivoGen, France)) supplemented with 20 ng/ml recombinant human epidermal growth factor (EGF) (Merck). Myometrial SMCs and endometrial stromal cells were maintained in complete culture media in monolayers to up to 70% confluency prior to seeding into microfluidic devices. For all co-/tri-cultures containing epithelial cells, media was always supplemented with 20 ng/ml EGF.

### Microfluidic device fabrication

Multi-layered microfluidic devices were fabricated using standard soft lithography techniques, following established protocols (Mulholland *et al.*, 2018; Paterson et al., 2022). A device consisted of up to 24 microfluidic channels, each of which was connected by two open wells, with channels hosting one or three arrays of 25 square microwells (250 × 250 × 200 µm). Briefly, a 10:1 ratio of poly(dimethylsiloxane) (PDMS) prepolymer (Sylgard 184, Dow Corning) to curing agent was mixed and dispensed onto patterned silicon wafers. The wafers were degassed and subsequently incubated at 85°C for a minimum of 3 h to allow curing of the PDMS solution. PDMS layers were then cut from the wafers and open wells were formed using a surgical biopsy punch (Miltex). PDMS layers were cleaned and treated with oxygen plasma (Pico plasma cleaner, Diener electronic, Germany) to permanently bond the layers together, after which a 1% solution of Synperonic^®^ F108 (Sigma Aldrich, UK) was injected to create ultra-low adhesion conditions. Subsequently, devices were washed using phosphate-buffered saline (PBS) and used for cell culture.

### Cell seeding and culture in microfluidic devices

For seeding of single-cell suspension (SMCs and stroma) or small epithelial cell clusters into microfluidic channels, cells were resuspended to a concentration of 2–4 × 10^6^ cells/ml. Cell solution volumes (5 µl) were seeded in the microfluidic device and incubated for 5 min to ensure settling of the cells to the bottom of the microwells. Excess cells were removed from the inlet and outlet open wells, which were then filled with 40 µl culture medium. For co-/tri-cultures of epithelia, stroma and SMCs, cells were seeded sequentially as described below.

### Incorporation of Matrigel into the on-chip cultures

For injection of 100% Matrigel^®^ Basement Membrane Matrix (# 356237 Corning, UK), cell culture media was removed from the inlet and outlet and the device placed between two cool packs for 10 min. 20 µl of Matrigel^®^ was then injected into the inlet, avoiding any air bubble formation. The device was further incubated for 15 min at 4°C before injecting 30 µl of ice-cold media to remove Matrigel^®^ from the channel above the microwells. After the media flow had stopped, the inlet and outlet were completely emptied again and 40 µl of cell culture media simultaneously injected into both. The device was incubated for 30 min at 37°C to allow complete gelation of Matrigel^®^.

For the injection of 5% Matrigel^®^, the appropriate volume of Matrigel^®^ was mixed into ice-cold medium. Cell culture media from inlet and outlet was fully removed and 5 µl of the 5% Matrigel^®^ solution injected into the inlet, followed by a 5 min incubation. The same process was repeated with the outlet, then both the inlet and outlet were topped up to 40 µl with the 5% Matrigel^®^ solution.

### Hormone responsiveness assay

Stromal cells and SMCs were thawed 72 h prior to seeding into microfluidic devices. Epithelial glands were thawed and digested on the day of seeding. Stromal and epithelial cells were seeded together in a 1:1 ratio. After 24 h, SMCs were added to the existing co-culture and this tri-culture was cultured for 24 h, followed by exchange of the culture medium for medium supplemented with 5% [v/v] Matrigel^®^. All cell culture medium used subsequently was supplemented with 5% Matrigel^®^. 24 h before hormone treatment, the culture media was replaced with media containing 2% [v/v] charcoal-stripped (CS) FBS (Thermo Fisher Scientific). For hormone treatment, during the first 24 h of hormone exposure, cells were cultured in 2% CS-FBS DMEM/F12 containing 10 nM β-estradiol (E2, Merck) to mimic the end of the proliferative phase. The initial 24 h hormone treatment was followed by treatment with 10 nM E2, 100 nM medroxyprogesterone acetate (MPA, Sigma Aldrich) and 500 µM cyclic adenosine monophosphate (cAMP, Merck). Conditioned medium was collected and replaced every two days over the course of six days by performing a 50% volume exchange (20 µl) with new hormone-containing media. All hormone treatments were conducted in three technical replicates.

### Enzyme-linked immunosorbent assay

IGFBP-1 and osteopontin concentrations were measured from conditioned medium using a human IGFBP-1 DuoSet sandwich ELISA kit (R&D Systems, UK) and human osteopontin enzyme-linked immunosorbent assay (ELISA) kit (ThermoFisher Scientific), respectively, according to the manufacturer’s protocols. Standard curves were generated using seven point, 2-fold serial dilutions and samples were assayed in duplicate. Absorbance was measured at 450 and 570 nm using a FLUOstar Omega microplate reader (BMG Labtech, UK). Optical imperfections were corrected by deducting the 570 nm value. Standard curves were generated using a four-parameter logistic regression model in GraphPad Prism Version 9.0.

### Cell labelling using cell trace dyes

To distinguish between different cell types by live-cell imaging, stroma, epithelia and SMCs were labelled with CellTrace dyes (InvitroGen, Thermo Fisher Scientific). Stromal cells were labelled as described by the manufacturer with CellTrace Far Red (CTFR), 72 h after thawing. SMCs were labelled with carboxyfluorescein succinimidyl ester (CFSE) (green) using the same dye concentrations. The epithelial cells were labelled in suspension with CellTrace Violet (CTV) immediately after the digestion of the glandular fraction.

### Immunocytochemistry (ICC)

Monolayer cell cultures were fixed with 4% [w/v] paraformaldehyde (PFA, Thermo Fisher Scientific) for 25 min, quenched with 100 mM glycine, and permeabilized with 0.2% [v/v] Triton X-100 in PBS for 10 min before blocking with 2% [w/v] bovine serum albumin (BSA, Merck) in PBS for 30 min. Afterwards, cells were incubated with primary or conjugated-primary antibodies for 24 h at 4°C, with secondary antibody incubations being 120 min long. Staining of 3D cell cultures in microfluidic devices was performed as described by Pettee *et al.* (2019). In brief, cells were washed with PBS, fixed with 4% PFA, permeabilized with 0.5% Triton X-100/PBS and blocked with 3% BSA. Primary antibodies were incubated for up to 48 h and secondary for another 24 h at 4°C in the dark before imaging. The antibodies used in the work were: anti-pan-cytokeratin (p-CK)-Alexa647 (SC8018AF647) 1:100 dilution (Santa Cruz, USA); anti-calponin-1 (CAL)-Alexa 488 (SC-58707AF488) 1:50 dilution (Santa Cruz); anti-smooth muscle actin (SMA)-Cy3 (C6198) 1:100 dilution (Merck); anti-vimentin (SC-6260) 1:50 dilution (Santa Cruz); anti-vimentin (ab45939); 1:200 dilution (Abcam); anti-CD324 (E-Cadherin) 1:200 dilution (eBioscience, Invitrogen); anti-estrogen receptor beta (sc-390243) 1:200 dilution (Santa Cruz Technologies); donkey anti-rabbit Alexa555 (Invitrogen); goat anti-rat Alexa488 (Invitrogen); goat anti-mouse Alexa647 (Invitrogen); and donkey anti-mouse-Alexa555 1:200 (Thermo Fisher Scientific).

### Calcium imaging

Cultures were loaded with a fluorescent calcium indicator using a 5 µM Cal-520™ AM (Stratech, UK) solution containing 0.02% [v/v] Pluronic F-127 and incubated for 60 min at 37°C followed by 15 min at room temperature in the dark. Prior to imaging, cells were washed with SFM and incubated for 20 min. Immediately before imaging, the inlet and outlet of the device were emptied. Changes in measured fluorescence levels in response to the addition of either endothelin-1 (ET-1, 100 nM) or oxytocin (OT, 100 nM) (both Merck) were then recorded (2.6 fps). The response to OT was measured first, followed by two washes with SFM and a 5 min rest period, before measuring the response to ET-1.

### Microscopy and image analysis

For Ca^2+^ imaging, an inverted Observer A1 microscope (Zeiss) with an Andor LucaR EMCCD camera and a ×10 0.25 NA objective was used. All other imaging was performed using an Observer Z1 (Zeiss) microscope, with an Apotome 2 structured illumination module used for optical sectioning (sections acquired every 7 µm in the vertical axis, moving up from the base of the 3D aggregate), connected to an Orca Flash 4.0 sCMOS camera (Hamamatsu) with an x20 0.8 NA, a ×10 0.3 NA or a ×5 0.16 NA objective. Data were analysed using either ZEN Blue version 3.4 (Zeiss) or ImageJ version 1.53f (NIH, USA). Data stacks obtained from Ca^2+^ imaging experiments were processed by first performing an eight-frame sequential subtraction (e.g. the pixel intensity values for each frame were subtracted from the values of the image eight frames ahead to produce an image where only changes in intracellular fluorescence levels are shown) and then applying a 3 × 3 median filter. Beginning at the onset of the response, the resulting images were averaged across three 20 s periods and the three images used to produce a colour map of responding cells that indicates variation in the response time of individual cells. Regions of interest (ROI) defining individual responding cells were manually drawn and maps of all viable cells were created from a 20-frame average of the baseline fluorescence prior to agonist application. To quantify overall dimensions and cell composition of individual 3D aggregates, fluorescent images were first thresholded in Zen Microscopy software (Zeiss) and then converted to binary images. These were analysed using a Matlab (Matlab R2023a) routine to extract area values. This procedure was performed to estimate both the overall aggregate dimensions (merging all fluorescent channels into the same binary image), and for each individual cell type (using individual fluorescent channel separately). Pixel area values were then converted to µm^2^ according to the objective used during image acquisition.

## Results

### Microfluidic microwell array device facilitates the formation of multi-cell type 3D cultures

**Figure 1.**
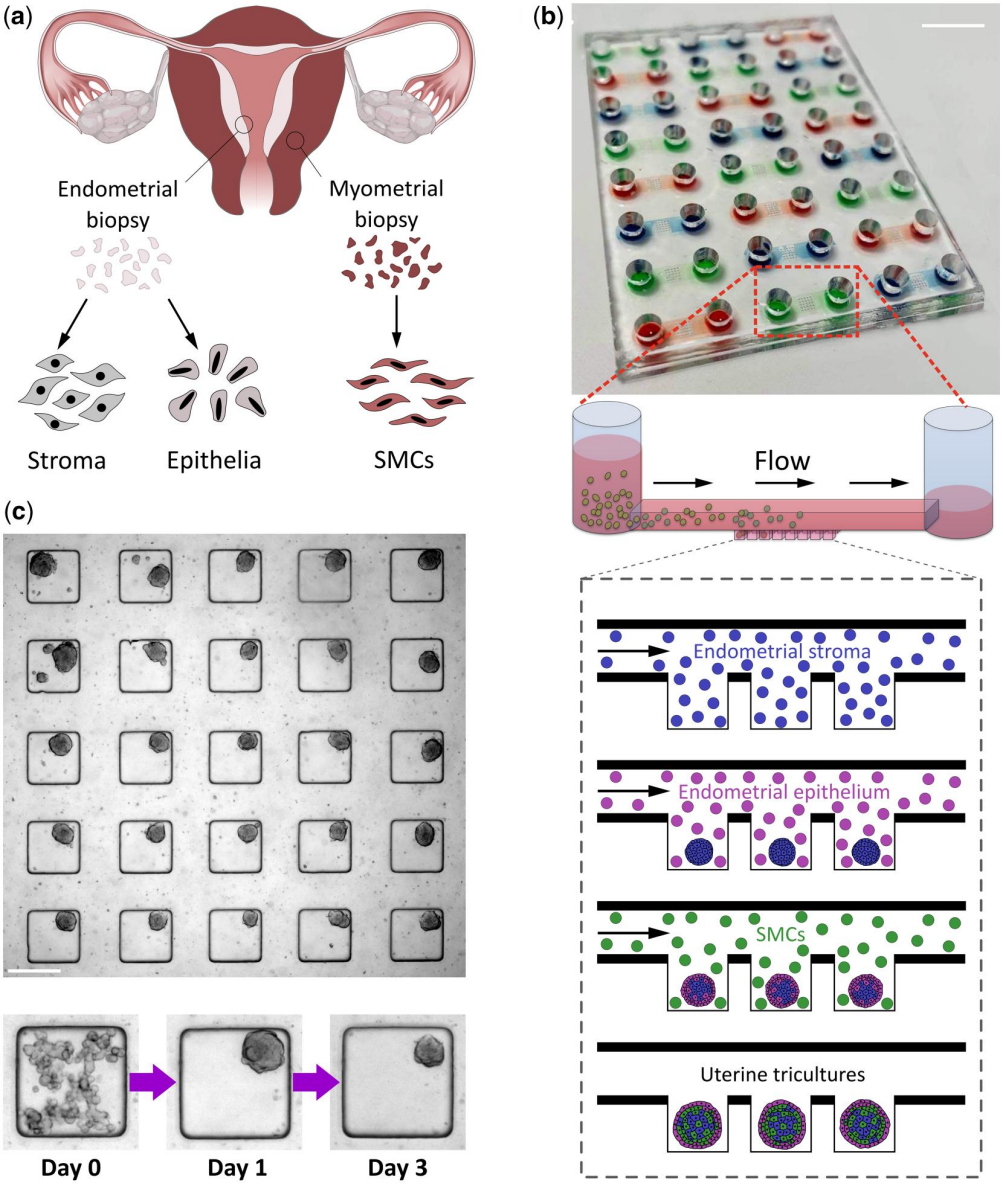
**On-chip approach to the creation of patient-derived 3D uterine models.** (**a**) Three separate cell fractions (epithelia and stroma from dissected endometrial tissue and smooth muscle cells from myometrium) are produced from biopsy tissue by a combination of enzymatic and mechanical digestion. (**b**) These fractions are sequentially seeded (by injection) into the microfluidic device, at the base of which is a microwell array, into which cells sediment and subsequently aggregate. Scalebar is 1 cm. (**c**) An example of a microwell array seeded with SMCs that aggregate to form 3D cell cultures in the non-adherent microwells. Scale bar is 250 µm.

Three cellular fractions (Fig. 1a) (endometrial epithelial cells, endometrial stromal cells and myometrial SMCs) were isolated from patient tissue and used in a range of different seeding scenarios to create multicellular 3D cultures. The creation and multiplexing of these cultures were facilitated by use of a microfluidic microwell array device, which enables the simultaneous formation of multiple individual 3D cultures. The device architecture (Fig. 1b) consists of 5 × 5 microwell arrays at the base of a microfluidic channel, accessed via open loading wells for straightforward cell injection. When a cell suspension is pipetted into one open well, the resulting capillary pressure difference creates a flow through the microfluidic channel, with cells being allowed to sediment into the microwells (Mulholland *et al*, 2018; Paterson *et al.*, 2022). The ultra-low adhesion surface of the device promoted the formation of 3D cellular aggregates within 24–48 h inside the microwells (Fig. 1c, showing the formation of myometrial spheroids), with no cells able to attach to the inner device surface. Sequential cell loading, washing and incubation stages could then be used to create multi-layered 3D cell aggregates.

### Tri-culture uterine wall model demonstrates a unique cellular organization

**Figure 2.**
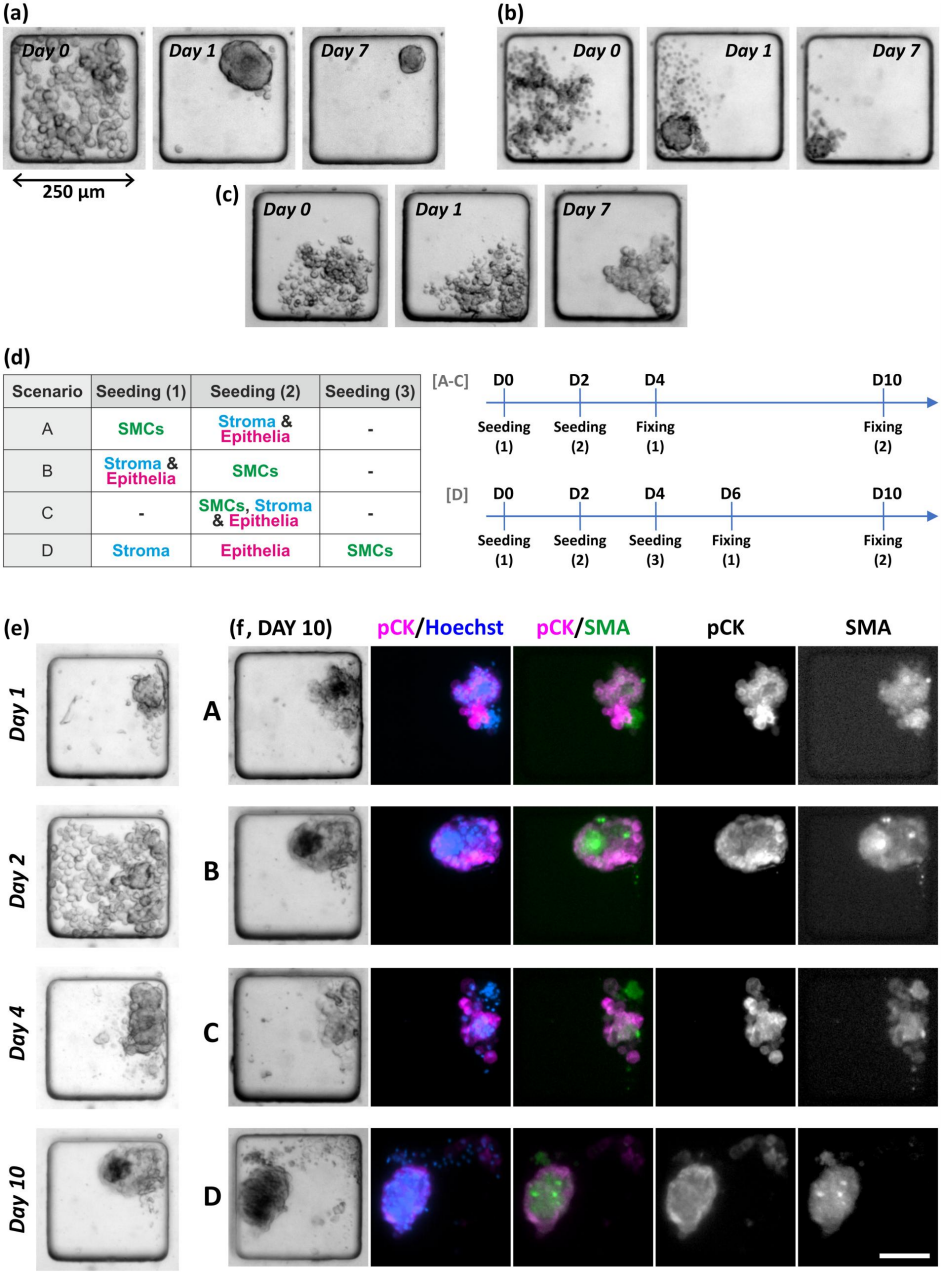
**Sequential seeding strategies for the creation of 3D uterine wall models.** Whilst smooth muscle (**a**) and stromal (**b**) cells readily aggregated to form compact spheroids, cells from the epithelial fraction alone (**c**) aggregated to form a looser, sheet-like structure. (**d**) The series of sequential seeding strategies for the formation of uterine tri-cultures investigated (Scenarios A, B, and D), alongside the simultaneous seeding of all three cell types to investigate their self-organization (Scenario C). (**e**) An example timeline from a sequential seeding scenario (Scenario B) illustrating cellular aggregation and organization at different time-points. (**f**) The cellular organization of the tri-cultures, at Day 10, resulting from the different seeding scenarios (A–D as marked) as assessed by staining for epithelial (pan-cytokeratin (pCK), magenta) and smooth muscle (smooth muscle actin (SMA), green) markers, alongside nuclear staining (Hoechst, blue). Scale bar is 100 µm.

We first characterized the 3D culture of individual cell types (Fig. 2a–c). The SMC and endometrial stroma single cell suspensions aggregated within 24 h to form compact spheroids, whilst the endometrial epithelial cells formed a looser structure. To prevent the epithelium from becoming diffuse, supporting cells were needed; when in co-culture with stromal cells, the epithelial cells either wrapped around the stromal cell spheroids or formed a distinct compact cluster attached to the aggregated stromal cells (Supplementary Fig. S1, with the markers used in all experiments validated in 2D culture as shown in Supplementary Fig. S2). To explore the formation of a uterine wall-like tri-culture model, a range of cell seeding strategies were investigated, sequentially or simultaneously seeding the different cell fractions as described in Fig. 2d, with an example time-course of cell aggregation shown in Fig. 2e. In addition, Supplementary Movie S1 (a time-lapse recording of fluorescently labelled cells) illustrates the aggregation of SMCs around a pre-formed epithelial-stromal cluster.

The different seeding order did not affect the overall pattern of cellular organization and broadly similar organization was observed for all sequences investigated. Although there was noticeable variation between individual cultures for any given protocol, in most cases the epithelia either formed the outer layer of the 3D cultures (Fig. 2f) or segregated into a separate cluster within a 3D aggregate (Supplementary Fig. S3a). Surprisingly, the most robust uterine mimics, that is a single compact 3D aggregate characterized by high cell viability (Supplementary Fig. S4) with a clear outer epithelial layer and SMC core, were produced when SMCs were seeded last (i.e. scenarios B and D). The largest number of these models was found with scenario D (where approximately 50% of 3D cultures were either wholly or partially surrounded by an epithelial layer). The use of the microwell array format facilitated the assessment of the phenotype of individual cultures, allowing the observation of rarer alternative 3D architectures (Supplementary Fig. S3). For example, with seeding scenario B (initially seeding with endometrial stroma/epithelia followed by SMCs), there were infrequent (2 out of 97 cultures, fixed across Day 6–10) examples of 3D cultures with an epithelial core surrounded by myometrial cells (Supplementary Fig. S3c). No examples of this architecture were seen in any other seeding scenario.

### Incorporation of a low-viscosity extracellular matrix support facilitates uterine-like organization

**Figure 3.**
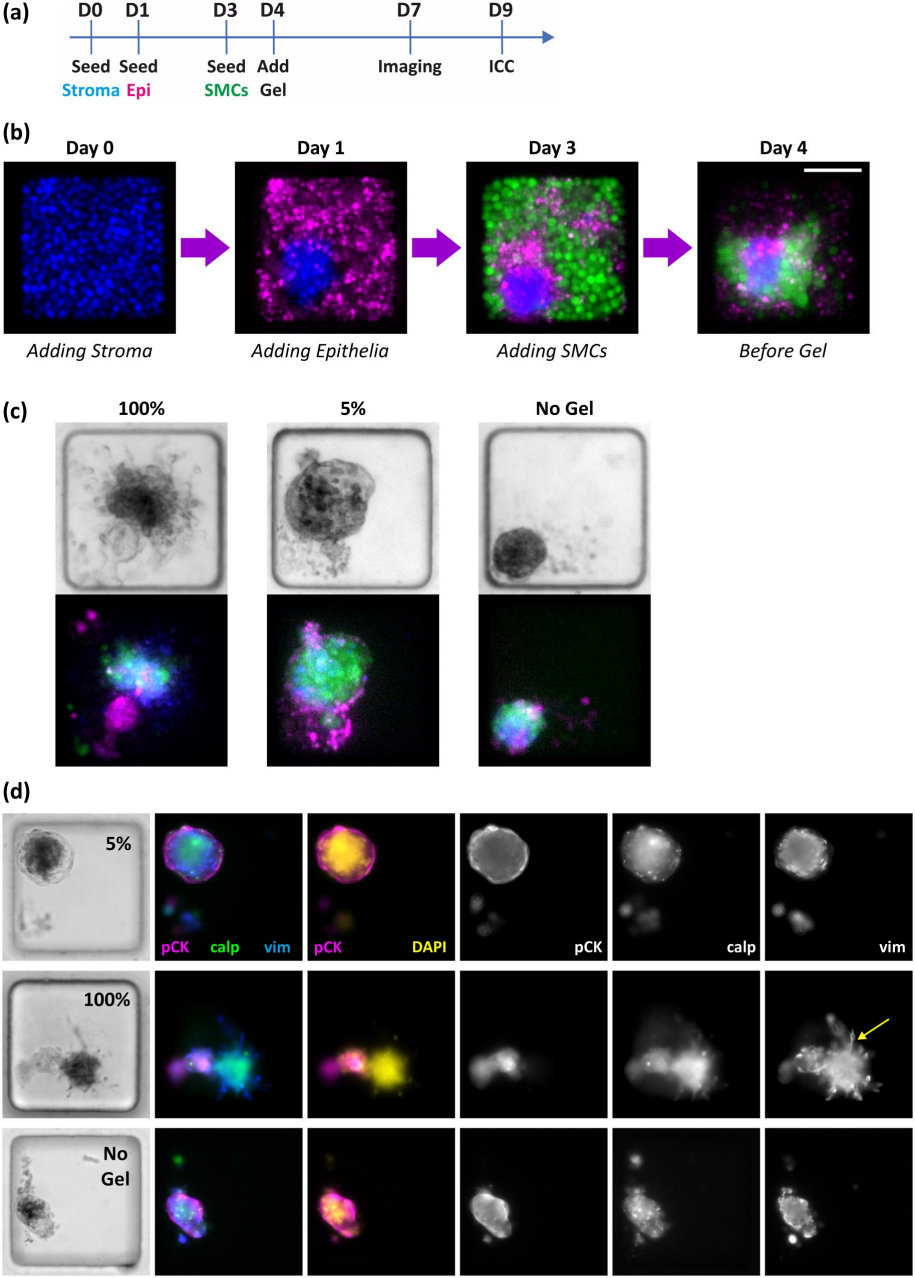
**Incorporation of low-viscosity ECM facilities the formation of 3D tri-cultures with uterine-like organization.** (**a**) The experimental timeline used for forming gel-supported 3D cultures. (**b**) Example time course, showing cells labelled with CellTrace™ reagents (stromal fraction, blue; epithelial fraction, magenta; smooth muscle cells (SMCs), green), illustrating cellular organization prior to gel addition (scale bar corresponds to 100 µm). (**c**) The different cellular architectures obtained on day 7 following the addition of either 100% Matrigel^®^, 5% Matrigel^®^ or no gel (cells labelled with CellTrace™ reagents, colours as noted in b). (**d**) Staining of 3D cultures on day 10 to determine the organization of the epithelial cells (pan-cytokeratin (pCK), magenta), stromal cells (vimentin, blue) and SMCs (calponin, green) in response to 5%, 100%, or no Matrigel^®^ conditions (DAPI staining shown in yellow). Note the stromal cell migration (yellow arrow) in the 100% gel example (observed in 90% of cultures). All microwells are 250 µm wide.

To ascertain whether the provision of extracellular matrix (ECM) proteins promotes the growth of robust 3D cultures with uterine-like organization and enhanced reproducibility, the incorporation of a solid gel support and of a low-viscosity matrix (both Matrigel^®^, 100% and 5% gels, respectively) were investigated. The same order of cell addition as in scenario D was used and live-cell fluorescence labelling was incorporated to better monitor cellular organization (Fig. 3a and b). The incorporation of 100% Matrigel^®^ did not result in the formation of uterine-like 3D models (Fig. 3c and d), rather it promoted separation of cell types and pronounced outgrowth of stromal cells, with clear signs of stromal cell migration into the Matrigel^®^ in nearly all cases (90% of cultures from day 9 immunocytochemistry; see also Supplementary Fig. S5c). However, the 5% Matrigel^®^ condition (Fig. 3c and d) was highly effective in promoting the formation of 3D uterine-like models, resulting in a clearly polarized cell distribution with distinct outer epithelial layers. These improved 3D cultures are particularly apparent when comparing an array incorporating the 5% gel to an array without the gel (comparing Supplementary Fig. S5a and b, both have the same cell seeding and imaging timeline).

### Variation of 3D culture properties with different patient samples and seeding scenarios: comparing culture size distribution and the extent of encapsulation

**Figure 4.**
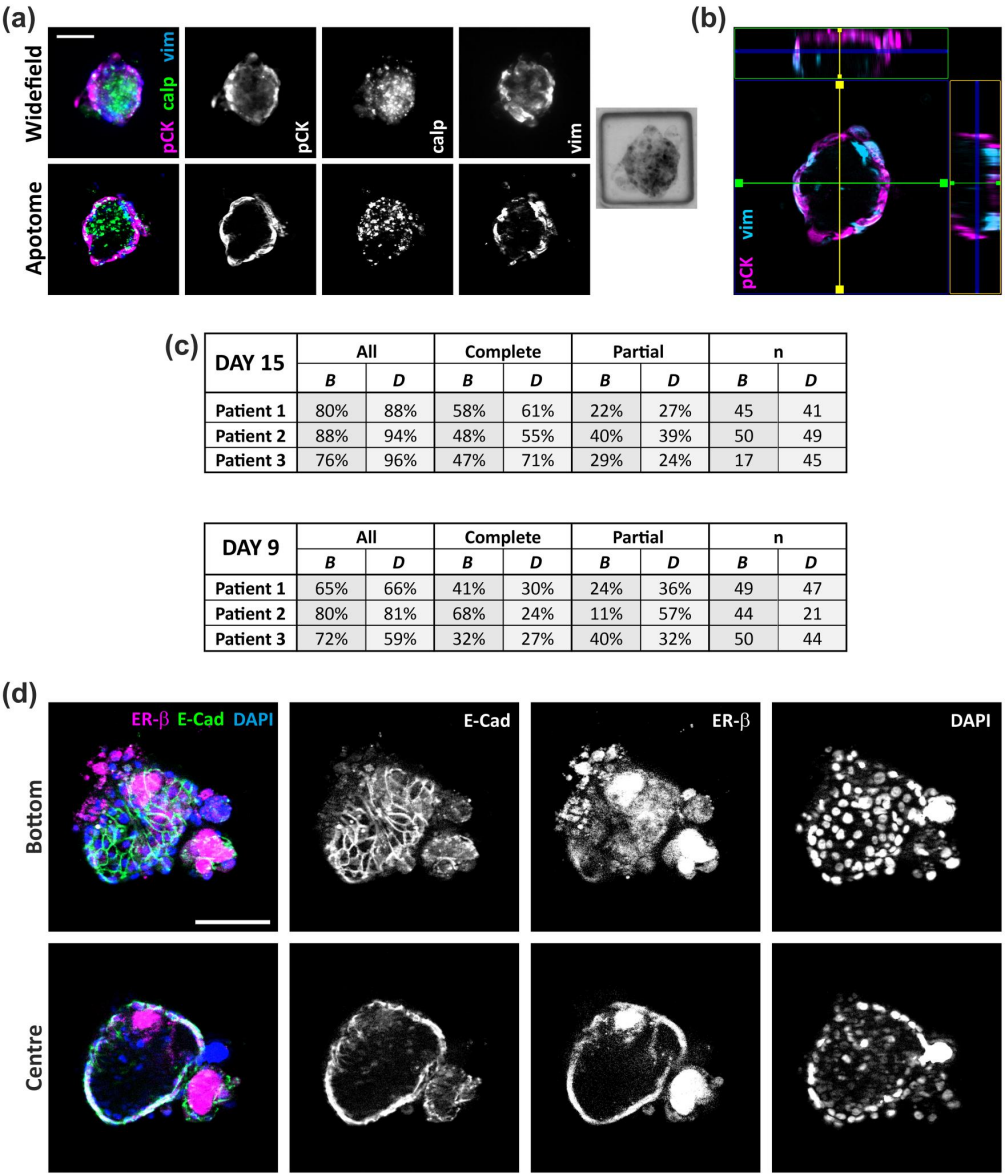
**Validation and quantification of epithelial encapsulation and expression of functional markers.** (**a**) Confirmation of epithelial encapsulation by optical sectioning (Apotome images shown in lower panels; upper panels showing standard wide-field imaging). (**b**) 3D reconstruction of the lower half of a 3D culture, showing *x*–*z* (green box) and *y*–*z* (yellow box) planes. (**c**) Quantification of the extent of encapsulation resulting from three different patient biopsies and two different seeding scenarios (n refers to the number of individual 3D cultures assessed). Day 15 samples were subjected to 6 days of hormonal stimulation, Day 9 samples were not stimulated. (**d**) Expression of the junctional marker E-cadherin (E-Cad) and oestrogen receptor beta (ER-beta (Day 15) cultures). Images obtained by optical sectioning, with the upper panels showing an optical section through the base of the 3D culture and lower panels a section through the centre. All scale bars correspond to 100 µm.

Having identified the key parameters required for obtaining 3D cultures with a uterine-like organization, i.e. the use of a low viscosity ECM support and the seeding of SMCs last, we further quantified important features of the resulting cultures: epithelial encapsulation, culture size, and expression of functional epithelial and stromal markers. Epithelial encapsulation was confirmed by optical sectioning (Apotome imaging in Fig. 4a and b and Supplementary Fig. S6). When comparing wide-field and Apotome imaging, epithelial encapsulation can be clearly identified by wide-field imaging, which allows for faster assessment of entire arrays containing cultures of different dimensions.

The distribution of individual culture dimensions reflects a range of parameters, including the precise cell seeding distribution of each cell type across an array and the cell growth rates. Individual aggregate dimensions were quantified (Supplementary Fig. S7) and compared across three different patient donors (three different donors for endometrial tissue, with a common myometrial donor) and for the two seeding scenarios, where SMCs were added last (B and D). This was assessed for cultures grown with (fixed on Day 15) and without (fixed on Day 9) hormonal stimulation that mimicked the secretory phase of the menstrual cycle. Differences in the mean values of the estimated diameter of the aggregates were observed between the two scenarios. With hormone stimulation and a 15-day growth period, the diameter was larger for Scenario D than for Scenario B for all donors, though this was not significant in the case of patient 1 (*P* = 0.953/0.013/<0.001 for patient 1/2/3; two-sample *t*-test). The opposite trend was observed for the non-stimulated cultures assessed at Day 9, where the Scenario B cultures had larger mean equivalent diameters (significantly different for patient 1 and 2; *P* = 0.021/0.005/0.051 for patient 1/2/3).

The percentage of individual cultures encapsulated with epithelium was then assessed for the same conditions. The smallest cultures, those with effective diameters ≤70 µm (6–8% of total cultures for Day 9/15), were excluded from the analysis (as their central focal planes were much lower than the majority of cultures being imaged and, therefore, difficult to accurately classify). Because some microwells contained more than one 3D cell aggregate, two levels of encapsulation were considered: ‘Complete’, where any 3D aggregate within the well was encapsulated; and ‘Partial’, for wells where two 3D aggregates were present and the larger of the two was encapsulated. In Fig. 4c, ‘All’ refers to the sum of Complete (%) and Partial (%) cases and shows that high levels of encapsulation were obtained, particularly following hormone stimulation (Day 15, >80%), where the highest percentages were obtained with Scenario D. There was not, however, a significant difference in encapsulation between the two seeding scenarios (*P* = 0.058/0.661, two-sample *t*-test for ‘All’ Day 15/Day 9, respectively). A visual comparison of cultures obtained from these three patients can be seen in Supplementary Fig. S8. When assessing a metric for the fraction of epithelial cells in a culture (Supplementary Fig. S9), with respect to the total cell population, no clear trend was identified when comparing the two different seeding scenarios across the three patients. However, the variation in the data for scenario D was significantly lower than for scenario B (*P* = 0.018, two-sample *t*-test comparing the variances obtained for each scenario, n = 3 patients).

With hormonal stimulation, which is required to demonstrate model functionality, Scenario D appeared to be most consistent. Therefore, we investigated the expression of both the epithelial junctional marker E-Cadherin, which plays a key role in epithelial barrier function, and oestrogen receptor (ER-beta), which is expressed by both stromal and epithelial cells in the uterus (Hapangama *et al.*, 2015). Hormone-stimulated Day 15 cultures exhibited E-cadherin expression at cell-cell junctions (visible in bottom plane) and ER-beta staining in the outer (endometrial) layers of the 3D culture (Fig. 4d).

### Uterine tri-cultures exhibit a decidualization response in the presence of ovarian hormones

**Figure 5.**
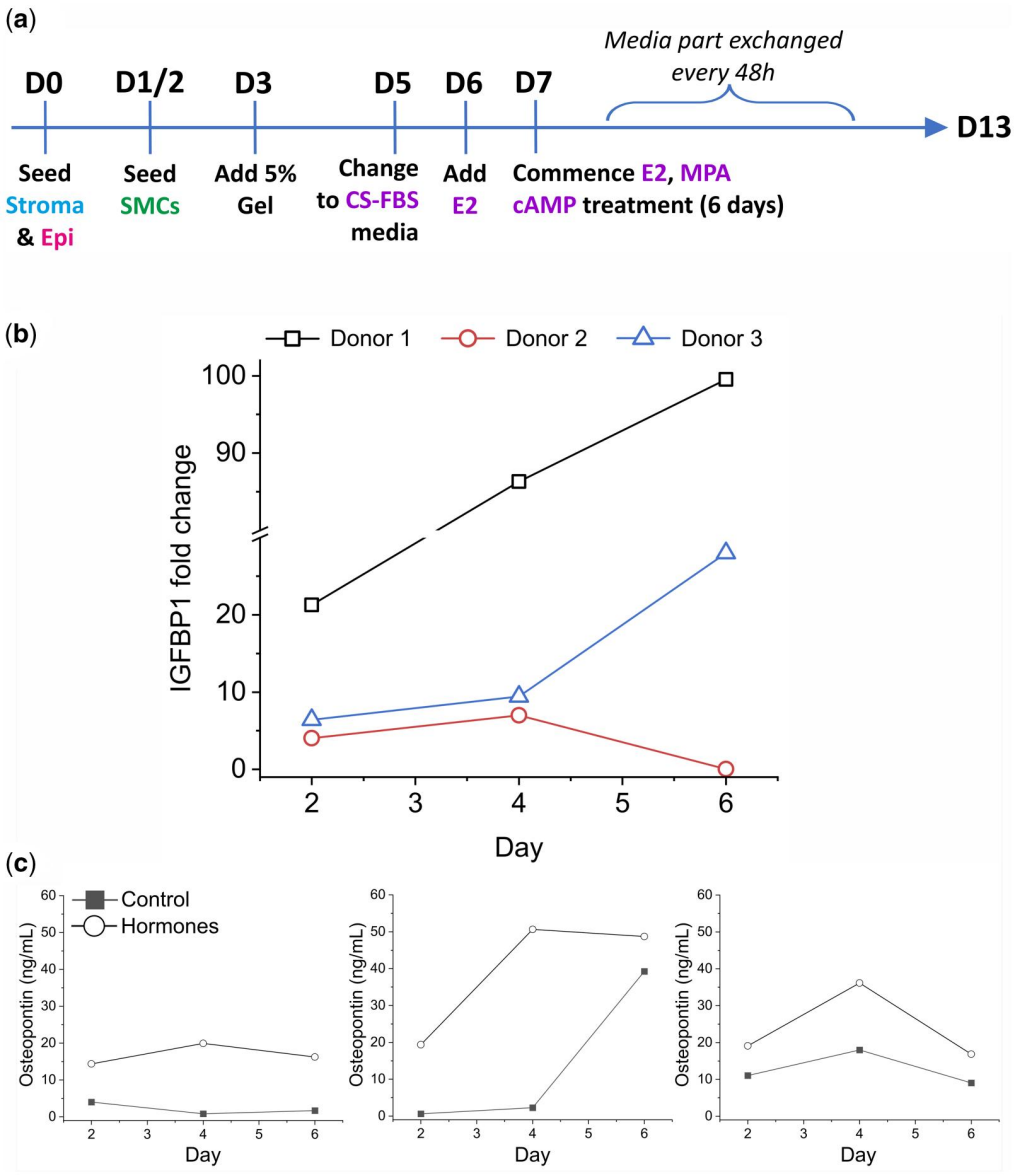
**The decidualization response of the 3D uterine wall models.** (**a**) Timeline of the hormone stimulation workflow. (**b**) IGFBP-1 secretion in response to hormone stimulation as measured by ELISA. Data are expressed as fold change relative to untreated controls. (**c**) Osteopontin secretion by hormone treated and control tricultures derived from three donors.

To assess the functionality of our microfluidic tri-culture models, secretion of the decidualization markers insulin-like growth factor binding protein-1 (IGFBP-1) and osteopontin were measured in response to hormonal stimulation. The 3D cultures with 5% Matrigel^®^ support were produced using cells isolated from secretory phase patient tissue and stimulated as noted above (seeding and stimulation timeline used here shown in Fig. 5a). In response to hormonal stimuli, IGFBP-1 secretion was observed to increase over time for two of the three donors (relative to untreated controls, Fig. 5b), with the third showing a response up to day 4. Osteopontin secretion was consistently higher in hormone treated tricultures compared to controls (Fig. 5c).

### Myometrial Ca^2+^ responses to stimulation with endothelin-1 and oxytocin

**Figure 6.**
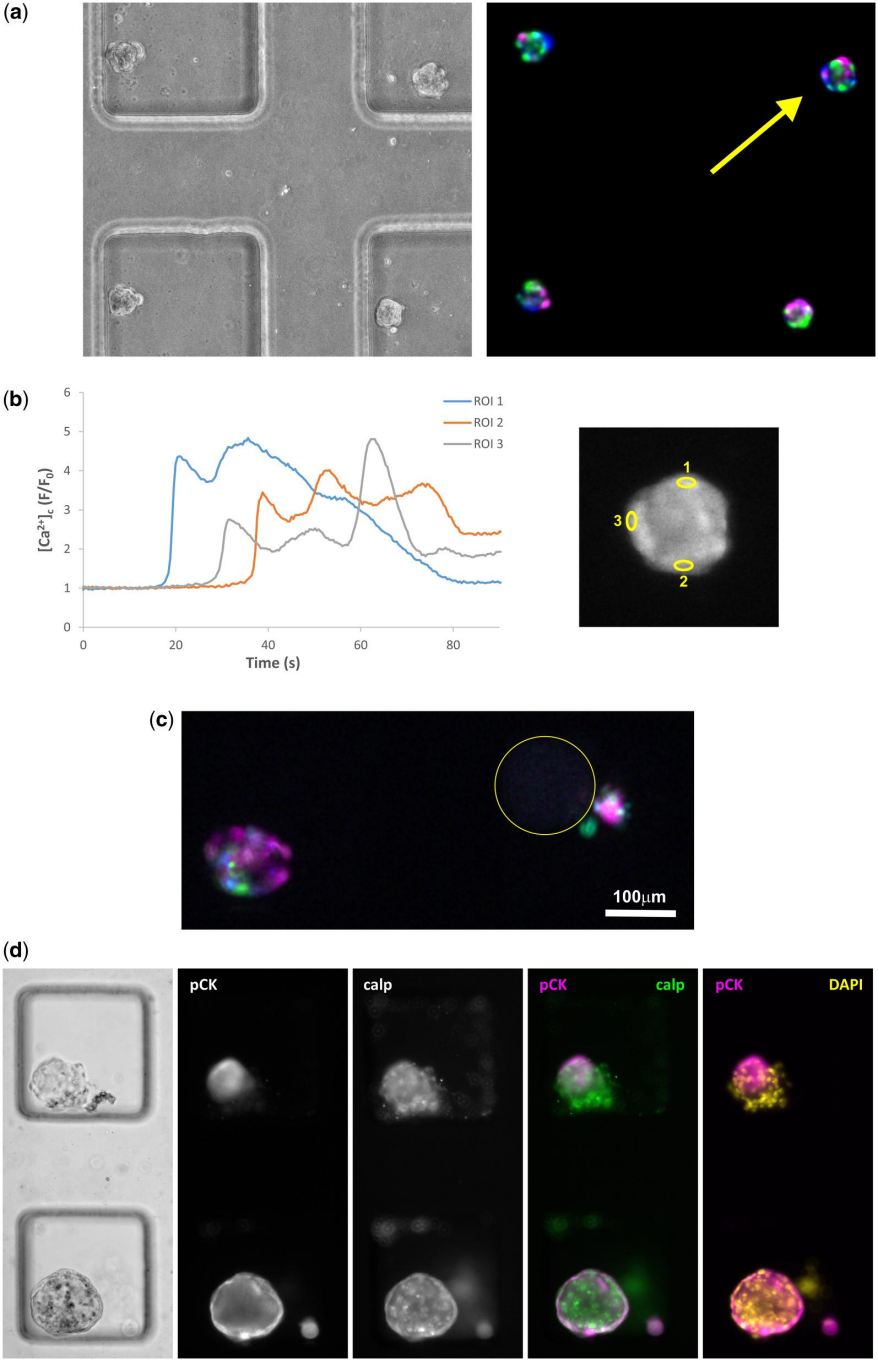
**Calcium responses of 3D uterine models to stimulation with endothelin-1 (ET-1).** (**a**) Smooth muscle cell (SMC)-only cultures responded strongly to stimulation with ET-1, with the majority of cells exhibiting a rise in intracellular calcium. The colour coding in the map of responding cells (right, with left showing bright-field image) indicates temporal variations in the response time of individual cells, grouping cells according to whether they responded in the first (magenta), second (green) or third (blue) 20 s period after the onset of the response. (**b**) Example traces illustrating the temporal response of individual cells within the 3D culture indicated by the yellow arrow in (a), with the position of the specific cells tracked indicated by the regions of interest (ROIs) highlighted in the right-hand image. (**c**) Map of responders for two 3D tri-cultures supplemented with 5% Matrigel^®^, with the left-hand culture responding strongly to ET-1 and the right-hand culture showing no response, aside from a small cluster of cells that were loosely attached to the main 3D culture (the position of latter is highlighted by the yellow circle). (**d**) Bright-field and immunofluorescence images for cultures in (c), with the responding culture at the top. The latter shows segregation of the epithelial cells (pCK) within the 3D structure, whilst the non-responding culture was encircled by an epithelial layer.

Further functional analysis was performed by assessing the calcium response of SMCs within the 3D cultures to stimulation with the agonists ET-1 and OT, measuring fold changes in cytoplasmic calcium concentration ([Ca^2+^]_c_) by fluorescence microscopy. When 3D cultures were formed using SMC fractions only, all cultures recorded (n = 15) showed a clear response to ET-1 stimulation (Fig. 6a and b), with a rise in [Ca^2+^]_c_ for most individual cells. However, when the optimized uterine tri-culture models were formed (using 5% Matrigel^®^), a different picture emerged (Fig. 6c and d). Clear responses to ET-1 were observed for 3D cultures that lacked an encircling outer epithelial layer but, in contrast, no responses were observed from larger cultures with an apparently complete outer epithelial layer. When compared to ET-1, the responses obtained to OT stimulation were substantially lower, in terms of the number of individual cells responding within a 3D culture (4 out of 12 SMC-only cultures showing >3 responding cells; 11 showing ≥1 cells), with a comparison between the two shown in Supplementary Fig. S10a. Interestingly, monolayer SMC cultures showed a stronger response to OT (with the majority of cells responding) than to ET-1 (with the minority of cells responding), as demonstrated in Supplementary Fig. S10b.

## Discussion

Model systems are important for understanding human physiology and pathologies, while being essential for mechanistic studies and drug development. There are inherent limitations to existing *ex vivo* and *in vitro* experimental approaches of studying human uterine physiology and prevailing pathologies. These include animal models not accurately recapitulating human reproductive physiology, simplified systems (2D models) being unable to resemble the complexity of *in vivo* cell–cell interactions, cell line-based models that are less relevant to non-transformed cells present in benign pathologies, and 3D models disregarding the significance of the EMJ and containing only endometrial components. To overcome these limitations, we have established a novel 3D uterine microfluidic tri-culture model using biopsy-derived cells and comprising both the myometrial and endometrial cellular participants of the uterine wall; thus, our model shows improved physiological relevance when compared with many existing models.

Recent studies in oncology have demonstrated the superior predictive power of biopsy-derived 3D models in assessing treatment response prior to clinical use (Snijder *et al.*, 2017; Vlachogiannis *et al.*, 2018). Our study fills the gap in the current literature, by exploiting a similar approach in devising a multi-cell type, patient-derived 3D model of the uterine wall that will be suitable and relevant for benign, chronic gynaecological conditions, where heterogeneity in disease presentation is well established (Tempest *et al.*, 2021; Maclean *et al.*, 2023; Powell *et al.*, 2023). In addition to improving physiological mimicry, for which the importance of using primary human cells has previously been highlighted in an endometrial model (Cook *et al*, 2017), our 3D model created from biopsy-derived cell populations would also enable patient-specific models of disease to be created, ultimately leading to personalized tailoring of therapies.

Microfluidic technology offers several advantages in miniaturization and multiplexing experimental capabilities, compared with standard culture techniques. The specific microfluidic methodology employed in this article enables control of sequential cell and reagent loading, maximizing the use of small patient tissue samples (which is particularly important when expanding cell numbers *in vitro* is not possible due to the potential loss of phenotype) and offering a medium throughput screening tool to test a range of therapeutic approaches using small quantities of patient-derived cells. This approach promotes observation of patient-to-patient differences with statistical relevance, enables the robust implementation of advanced culture protocols, and could ultimately facilitate disease modelling of individual patients and thus, personalized drug screening.

To create a robust 3D uterine wall model, with an outer epithelial layer enclosing an inner myocyte core, optimum results were obtained when SMCs were seeded last and by the inclusion of a low-viscosity ECM support (5% Matrigel^®^). Our observation agrees with Hirokawa *et al.* (2021), who previously reported that low viscosity ECM support facilitated the efficient establishment of large intestine organoids, removing the requirement for a solid matrix. Our use of a low viscosity ECM support to establish robust individual multicellular 3D structures with distinct uterine-like cellular organization is favourable to the study of endometrial physiology and contrasts to the use of a solid matrix (100% Matrigel^®^), where segregation of individual cell types and outward migration of stromal cells was pronounced.

Recent single cell studies have amply demonstrated human endometrial transcriptomic heterogeneity at the cellular level, in health and in pathologies, such as adenomyosis and endometriosis (Ma *et al.*, 2021; Goad *et al.*, 2022; Yildiz *et al.*, 2023). An additional benefit of a microwell array format, therefore, is the possibility to study distinct cell subtype-specific behaviours. Such functional differences can be readily identified when screening large numbers of cultures across multiple arrays. As an example, we observed rare instances of an epithelial core surrounded by a layer of myometrial cells, which is potentially useful in modelling the pathogenesis of adenomyosis (Gnecco *et al*, 2020). Identification of behaviours of less abundant cellular subtypes would improve the understanding of their specific contribution to disease pathogenesis and differential responses to medications.

To validate the physiological relevance of our model, two functional assays were performed. The first of these was a decidualization assay. Ovarian hormone-driven decidualization facilitates human pregnancy establishment by transforming the endometrium into a receptive and permissive environment for embryo implantation (Gellersen and Brosens, 2003), and cAMP-mediated protein kinase A (PKA) is also crucial for the decidualization process (Kusama *et al*, 2014). Triculture models derived from endometrial biopsies from three donors in the secretory phase of the cycle, demonstrated a decidual response in the presence of E2, MPA, and cAMP. Relative levels of secreted IGFBP-1 varied between donors, however, all three donor-derived tricultures demonstrated some recapitulation of response. The best decidualization response according to IGFBP-1 secretions was seen in the late secretory sample, whereas the weakest response was produced by the early secretory sample. This may demonstrate either the expected patient-specific heterogeneity in cellular response (Abbas *et al.*, 2020), or reflect the exact menstrual cycle phase-specific differences in response. Future work including multiple samples at different phases of the cycle from the same donor and scrutiny of a larger number of samples from different donors will be required to further confirm these possibilities. Osteopontin is expressed by the endometrial glandular epithelium during the secretory phase of the menstrual cycle and is regulated by progesterone (Johnson *et al.*, 2000; Apparao *et al.*, 2001; von Wolff *et al.*, 2004; Hapangama *et al.*, 2012). Tricultures consistently exhibited increased osteopontin secretion in the presence of hormones, thus demonstrating the expected epithelial differentiation response in uterine tricultures.

In 3D culture, the patient-derived myocytes showed strong responses to ET-1, compared with OT, a contrasting observation to the response observed in 2D culture of the same myocytes. Differences in gene and protein expression levels between 2D and 3D cultures of human myometrial cells have previously been reported, with Malik *et al.* (2014) demonstrating that the culture architecture alters cellular behaviour. The higher sensitivity of pregnant myometrial cells to OT compared with non-pregnant myometrium is also well-established (Arrowsmith *et al.*, 2012; Fuchs and Fuchs, 1963). Although we obtained myocytes from pregnant women, their response to OT was reduced with the addition of endometrial cellular components in 3D culture, indicative of adapting a non-pregnant phenotype. Furthermore, tri-cultures also demonstrated an architecture-dependent response. The lack of Ca^2+^ response to ET-1 in larger, uterine-like models, where myometrium is encapsulated with endometrial epithelium, may be a consequence of barrier function due to a complete epithelial layer with junction formation.

Although this model contains the main primary cell types constituting the uterine wall, endometrial cells demonstrate region-specific cellular differences. Therefore, future work is required to ascertain whether specific cells obtained from the endometrial sub-regions (e.g. basalis) are needed or whether endometrial tissue from any region will acquire properties of the basalis, which is adjacent to the myometrium *in vivo*, when co-cultured with myometrium. High-resolution imaging techniques, such as electron microscopy, as well as spatial transcriptomics or single cell sequencing, could be employed in future studies to further ascertain the functional relevance, organization and cellular phenotype of uterine tricultures.

Together, the results of morphological characterizations and functional assays presented validate this microfluidic triculture approach (using endometrial epithelial and stromal cells, and myocytes) for the creation of a physiologically relevant human model of uterine function, providing the first myocyte-containing, patient-derived organ-on-a-chip uterine model. Our human *in vitro* model provides a new tool that could be further developed and employed to improve our understanding of the cellular behaviours underlying chronic gynaecological disorders, such as endometriosis, adenomyosis and leiomyoma, with applications in personalized drug screening and the assessment of patient-to-patient heterogeneity in responses.

## Supplementary Material

deae214_Supplementary_Movie_S1

deae214_Supplementary_Figure_S1

deae214_Supplementary_Figure_S2

deae214_Supplementary_Figure_S3

deae214_Supplementary_Figure_S4

deae214_Supplementary_Figure_S5

deae214_Supplementary_Figure_S6

deae214_Supplementary_Figure_S7

deae214_Supplementary_Figure_S8

deae214_Supplementary_Figure_S9

deae214_Supplementary_Figure_S10

deae214_Supplementary_Table_S1

## Data Availability

The data that support the findings of this study are available upon reasonable request from the authors.

## References

[deae214-B1] Abbas Y , Brunel LG, Hollinshead MS, Fernando RC, Gardner L, Duncan I, Moffett A, Best S, Turco MY, Burton GJ et al Generation of a three-dimensional collagen scaffold-based model of the human endometrium. Interface Focus 2020;10:20190079. 32194932 10.1098/rsfs.2019.0079 PMC7061944

[deae214-B2] Ahn J , Yoon MJ, Hong SH, Cha H, Lee D, Koo HS, Ko JE, Lee J, Oh S, Jeon NL et al Three-dimensional microengineered vascularised endometrium-on-a-chip. Hum Reprod 2021;36:2720–2731. 34363466 10.1093/humrep/deab186 PMC8450871

[deae214-B3] Apparao KB , Murray MJ, Fritz MA, Meyer WR, Chambers AF, Truong PR, Lessey BA. Osteopontin and its receptor alphavbeta(3) integrin are coexpressed in the human endometrium during the menstrual cycle but regulated differentially. J Clin Endocrinol Metab 2001;86:4991–5000. 11600576 10.1210/jcem.86.10.7906

[deae214-B4] Arrowsmith S , Robinson H, Noble K, Wray S. What do we know about what happens to myometrial function as women age? J Muscle Res Cell Motil 2012;33:209–217. 22644420 10.1007/s10974-012-9300-2 PMC3413813

[deae214-B5] Campo H , Murphy A, Yildiz S, Woodruff T, Cervello I, Kim JJ. Microphysiological modeling of the human endometrium. Tissue Eng Part A 2020;26:759–768. 32348708 10.1089/ten.tea.2020.0022 PMC7398432

[deae214-B6] Cook CD , Hill AS, Guo M, Stockdale L, Papps JP, Isaacson KB, Lauffenburger DA, Griffith LG. Local remodeling of synthetic extracellular matrix microenvironments by co-cultured endometrial epithelial and stromal cells enables long-term dynamic physiological function. Integr Biol (Camb) 2017;9:271–289. 28317948 10.1039/c6ib00245e PMC5461964

[deae214-B7] Fuchs AR , Fuchs F. Spontaneous motility and oxytocin response of the pregnant and non-pregnant human uterine muscle *in vitro*. Studies on the contractility of the human myometrium. J Obstet Gynaecol Br Commonw 1963;70:658–664. 14078042 10.1111/j.1471-0528.1963.tb04962.x

[deae214-B8] Goad J , Rudolph J, Zandigohar M, Tae M, Dai Y, Wei J-J, Bulun SE, Chakravarti D, Rajkovic A. Single-cell sequencing reveals novel cellular heterogeneity in uterine leiomyomas. Hum Reprod 2022;37:2334–2349. 36001050 10.1093/humrep/deac183 PMC9802286

[deae214-B9] Gellersen B , Brosens J. Cyclic AMP and progesterone receptor cross-talk in human endometrium: a decidualizing affair. J Endocrinol 2003;178:357–372. 12967329 10.1677/joe.0.1780357

[deae214-B10] Gnecco JS , Brown AT, Kan EL, Baugh L, Ives C, Loring M, Griffith LG. Physiomimetic models of adenomyosis. Semin Reprod Med 2020;38:179–196. 33176387 10.1055/s-0040-1719084 PMC7803459

[deae214-B11] Gnecco JS , Ding T, Smith C, Lu J, Bruner-Tran KL, Osteen KG. Hemodynamic forces enhance decidualization via endothelial-derived prostaglandin E2 and prostacyclin in a microfluidic model of the human endometrium. Hum Reprod 2019;34:702–714. 30789661 10.1093/humrep/dez003 PMC6443116

[deae214-B12] Gołąbek-Grenda A , Olejnik A. *In vitro* modeling of endometriosis and endometriotic microenvironment—challenges and recent advances. Cell Signal 2022;97:110375. 35690293 10.1016/j.cellsig.2022.110375

[deae214-B13] Hao M , Liu X, Guo SW. Adenomyosis in mice resulting from mechanically or thermally induced endometrial-myometrial interface disruption and its possible prevention. Reprod Biomed Online 2020;41:925–942. 32921577 10.1016/j.rbmo.2020.07.023

[deae214-B14] Hapangama DK , Kamal AM, Bulmer JN. Estrogen receptor β: the guardian of the endometrium. Hum Reprod Update 2015;21:174–193. 25305176 10.1093/humupd/dmu053

[deae214-B15] Hapangama DK , Raju RS, Valentijn AJ, Barraclough D, Hart A, Turner MA, Platt-Higgins A, Barraclough R, Rudland PS. Aberrant expression of metastasis-inducing proteins in ectopic and matched eutopic endometrium of women with endometriosis: implications for the pathogenesis of endometriosis. Hum Reprod 2012;27:394–407. 22147918 10.1093/humrep/der412

[deae214-B16] Hertelendy F , Zakar T. Regulation of myometrial smooth muscle functions. Curr Pharm Des 2004;10:2499–2517. 15320759 10.2174/1381612043383926

[deae214-B17] Hirokawa Y , Clarke J, Palmieri M, Tan T, Mouradov D, Li S, Lin C, Li F, Luo H, Wu K et al Low-viscosity matrix suspension culture enables scalable analysis of patient-derived organoids and tumoroids from the large intestine. Commun Biol 2021;4:1067. 34518628 10.1038/s42003-021-02607-y PMC8438070

[deae214-B18] Jansen C , Kastelein AW, Kleinrouweler CE, Van Leeuwen E, De Jong KH, Pajkrt E, Van Noorden CJF. Development of placental abnormalities in location and anatomy. Acta Obstet Gynecol Scand 2020;99:983–993. 32108320 10.1111/aogs.13834 PMC7496588

[deae214-B19] Johnson GA , Spencer TE, Burghardt RC, Taylor KM, Gray CA, Bazer FW. Progesterone modulation of osteopontin gene expression in the ovine uterus. Biol Reprod 2000;62:1315–1321.. 10775182 10.1095/biolreprod62.5.1315

[deae214-B20] Kamal AM , Bulmer JN, DeCruze SB, Stringfellow HF, Martin-Hirsch P, Hapangama DK. Androgen receptors are acquired by healthy postmenopausal endometrial epithelium and their subsequent loss in endometrial cancer is associated with poor survival. Br J Cancer 2016;114:688–696. 26930451 10.1038/bjc.2016.16 PMC4800292

[deae214-B21] Kunz G , Beil D, Huppert P, Noe M, Kissler S, Leyendecker G. Adenomyosis in endometriosis—prevalence and impact on fertility. Evidence from magnetic resonance imaging. Hum Reprod 2005;20:2309–2316. 15919780 10.1093/humrep/dei021

[deae214-B22] Kuperman T , Gavriel M, Gotlib R, Zhang Y, Jaffa A, Elad D, Grisaru D. Tissue-engineered multi-cellular models of the uterine wall. Biomech Model Mechanobiol 2020;19:1629–1639. 31997029 10.1007/s10237-020-01296-6

[deae214-B23] Kusama K , Yoshie M, Tamura K, Nakayama T, Nishi H, Isaka K, Tachikawa E. The role of exchange protein directly activated by cyclic AMP 2-mediated calreticulin expression in the decidualization of human endometrial stromal cells. Endocrinology 2014;155:240–248. 24169561 10.1210/en.2013-1478

[deae214-B24] Leyendecker G , Wildt L. A new concept of endometriosis and adenomyosis: tissue injury and repair (TIAR). Horm Mol Biol Clin Investig 2011;5:125–142. 10.1515/HMBCI.2011.002 25961248

[deae214-B25] Li X , Kodithuwakku SP, Chan RWS, Yeung WSB, Yao Y, Ng EHY, Chiu PCN, Lee CL. Three-dimensional culture models of human endometrium for studying trophoblast-endometrium interaction during implantation. Reprod Biol Endocrinol 2022;20:120. 35964080 10.1186/s12958-022-00973-8 PMC9375428

[deae214-B26] Lü SH , Wang HB, Liu H, Wang HP, Lin QX, Li DX, Song YX, Duan CM, Feng LX, Wang CY. Reconstruction of engineered uterine tissues containing smooth muscle layer in collagen/matrigel scaffold *in vitro*. Tissue Eng Part A 2009;15:1611–1618. 19061433 10.1089/ten.tea.2008.0187

[deae214-B27] Ma J , Zhang L, Zhan H, Mo Y, Ren Z, Shao A, Lin J. Single-cell transcriptomic analysis of endometriosis provides insights into fibroblast fates and immune cell heterogeneity. Cell Biosci 2021;11:125. 34233737 10.1186/s13578-021-00637-x PMC8261960

[deae214-B28] Maclean A , Bunni E, Makrydima S, Withington A, Kamal AM, Valentijn AJ, Hapangama DK. Fallopian tube epithelial cells express androgen receptor and have a distinct hormonal responsiveness when compared with endometrial epithelium. Hum Reprod2020b;35:2097–2106. 32876325 10.1093/humrep/deaa177

[deae214-B29] Maclean A , Barzilova V, Patel S, Bates F, Hapangama DK. Characterising the immune cell phenotype of ectopic adenomyosis lesions compared with eutopic endometrium: A systematic review. J Reprod Immunol 2023;157:103925. 36870297 10.1016/j.jri.2023.103925

[deae214-B30] Maclean A , Kamal A, Adishesh M, Alnafakh R, Tempest N, Hapangama DK. Human uterine biopsy: research value and common pitfalls. Int J Reprod Med2020a;2020:9275360. 32411783 10.1155/2020/9275360 PMC7206876

[deae214-B31] Malik M , Britten J, Segars J, Catherino WH. Leiomyoma cells in 3-dimensional cultures demonstrate an attenuated response to Fasudil, a Rho-kinase inhibitor, when compared to 2-dimensional cultures. Reprod Sci 2014;21:1126–1138. 25084783 10.1177/1933719114545240 PMC4212347

[deae214-B32] Mulholland T , McAllister M, Patek S, Flint D, Underwood M, Sim A, Edwards J, Zagnoni M. Drug screening of biopsy-derived spheroids using a self-generated microfluidic concentration gradient. Sci Rep 2018;8:14672. 30279484 10.1038/s41598-018-33055-0 PMC6168499

[deae214-B33] Naftalin J , Jurkovic D. The endometrial–myometrial junction: a fresh look at a busy crossing. Ultrasound Obstet Gynecol 2009;34:1–11. 10.1002/uog.6432 19565525

[deae214-B34] Park Y , Jung JG, Yu ZC, Asaka R, Shen W, Wang Y, Jung WH, Tomaszewski A, Shimberg G, Chen Y et al A novel human endometrial epithelial cell line for modeling gynecological diseases and for drug screening. Lab Invest 2021;101:1505–1512. 34376780 10.1038/s41374-021-00624-3 PMC8720294

[deae214-B35] Paterson K , Paterson S, Mulholland T, Coffelt SB, Zagnoni M. Assessment of CAR-T cell-mediated cytotoxicity in 3D microfluidic cancer co-culture models for combination therapy. IEEE Open J Eng Med Biol 2022;3:86–95. 35813488 10.1109/OJEMB.2022.3178302 PMC9252335

[deae214-B36] Pettee KM , Becker KN, Alberts AS, Reinard KA, Schroeder JL, Eisenmann KM. Targeting the mDia formin-assembled cytoskeleton is an effective anti-invasion strategy in adult high-grade glioma patient-derived neurospheres. Cancers (Basel) 2019;11:392. 30897774 10.3390/cancers11030392 PMC6468841

[deae214-B37] Powell SG , Sharma P, Masterson S, Wyatt J, Arshad I, Ahmed S, Lash G, Cross M, Hapangama DK. Vascularisation in deep endometriosis: a systematic review with narrative outcomes. Cells 2023;12:1318. 37174718 10.3390/cells12091318 PMC10177118

[deae214-B38] Rawlings TM , Makwana K, Tryfonos M, Lucas ES. Organoids to model the endometrium: implantation and beyond. Reprod Fertil 2021;2:R85–R101. 35118399 10.1530/RAF-21-0023 PMC8801025

[deae214-B39] Snijder B , Vladimer GI, Krall N, Miura K, Schmolke AS, Kornauth C, Lopez de la Fuente O, Choi HS, van der Kouwe E, Gültekin S et al Image-based *ex-vivo* drug screening for patients with aggressive haematological malignancies: interim results from a single-arm, open-label, pilot study. Lancet Haematol 2017;4:e595–e606. 29153976 10.1016/S2352-3026(17)30208-9 PMC5719985

[deae214-B40] Tempest N , Hill CJ, Maclean A, Marston K, Powell SG, Al-Lamee H, Hapangama DK. Novel microarchitecture of human endometrial glands: implications in endometrial regeneration and pathologies. Hum Reprod Update 2022;28:153–171. 34875046 10.1093/humupd/dmab039 PMC8888994

[deae214-B41] Tempest N , Hill CJ, Whelan A, De Silva A, Drakeley AJ, Phelan MM, Hapangama DK. Symptomatology and serum nuclear magnetic resonance metabolomics; do they predict endometriosis in fertile women undergoing laparoscopic sterilisation? A prospective cross-sectional study. Reprod Sci 2021;28:3480–3490. 34524640 10.1007/s43032-021-00725-w PMC8580895

[deae214-B42] Valentijn AJ , Palial K, Al-Lamee H, Tempest N, Drury J, Von Zglinicki T, Saretzki G, Murray P, Gargett CE, Hapangama DK. SSEA-1 isolates human endometrial basal glandular epithelial cells: phenotypic and functional characterization and implications in the pathogenesis of endometriosis. Hum Reprod 2013;28:2695–2708. 23847113 10.1093/humrep/det285

[deae214-B43] Vidimar V , Chakravarti D, Bulun SE, Yin P, Nowak R, Wei JJ, Kim JJ. The AKT/BCL-2 axis mediates survival of uterine leiomyoma in a novel 3D spheroid model. Endocrinology 2018;159:1453–1462. 29381777 10.1210/en.2017-03191 PMC5839731

[deae214-B44] Vlachogiannis G , Hedayat S, Vatsiou A, Jamin Y, Fernández-Mateos J, Khan K, Lampis A, Eason K, Ian Huntingford I, Burke R et al Patient-derived organoids model treatment response of metastatic gastrointestinal cancers. Science 2018;359:920–926. 29472484 10.1126/science.aao2774 PMC6112415

[deae214-B45] von Wolff M , Bohlmann MK, Fiedler C, Ursel S, Strowitzki T. Osteopontin is up-regulated in human decidual stromal cells. Fertil Steril 2004;81:741–748. 15019804 10.1016/j.fertnstert.2003.08.027

[deae214-B46] Xiao S , Coppeta JR, Rogers HB, Isenberg BC, Zhu J, Olalekan SA, McKinnon KE, Dokic D, Rashedi AS, Haisenleder DJ et al A microfluidic culture model of the human reproductive tract and 28-day menstrual cycle. Nat Commun 2017;8:14584. 28350383 10.1038/ncomms14584 PMC5379057

[deae214-B47] Xie J , Xu X, Yin P, Li Y, Guo H, Kujawa S, Chakravarti D, Bulun S, Kim JJ, Wei JJ. Application of *ex-vivo* spheroid model system for the analysis of senescence and senolytic phenotypes in uterine leiomyoma. Lab Invest 2018;98:1575–1587. 30206313 10.1038/s41374-018-0117-5 PMC6265265

[deae214-B48] Yamaguchi M , Yoshihara K, Suda K, Nakaoka H, Yachida N, Ueda H, Sugino K, Mori Y, Yamawaki K, Tamura R et al Three-dimensional understanding of the morphological complexity of the human uterine endometrium. iScience 2021;24:102258. 33796844 10.1016/j.isci.2021.102258 PMC7995615

[deae214-B49] Yang C , Ikeda K, Horie-Inoue K, Sato W, Hasegawa K, Takeda S, Itakura A, Inoue S. Transcriptomic analysis of hormone-sensitive patient-derived endometrial cancer spheroid culture defines Efp as a proliferation modulator. Biochem Biophys Res Commun 2021;548:204–210. 33647797 10.1016/j.bbrc.2021.02.066

[deae214-B50] Yildiz S , Kinali M, Wei JJ, Milad M, Yin P, Adli M, Bulun SE. Adenomyosis: single-cell transcriptomic analysis reveals a paracrine mesenchymal–epithelial interaction involving the WNT/SFRP pathway. Fertil Steril 2023;119:869–882. 36736810 10.1016/j.fertnstert.2023.01.041 PMC11257082

