# Supplementary material for: Functional, patient-derived 3D tri-culture models of the uterine wall in a microfluidic array
Source: Hum Reprod. 2024 Sep 15;39(11):2537–50. doi: 10.1093/humrep/deae214 (PMC11532614; doi:10.1093/humrep/deae214)
Supplement: deae214_Supplementary_Figure_S1 [file deae214_supplementary_figure_s1.pdf]

Human Reproduction, 2024, 1–1
https://doi.org/10.1093/humrep/deae214
Supplementary Data

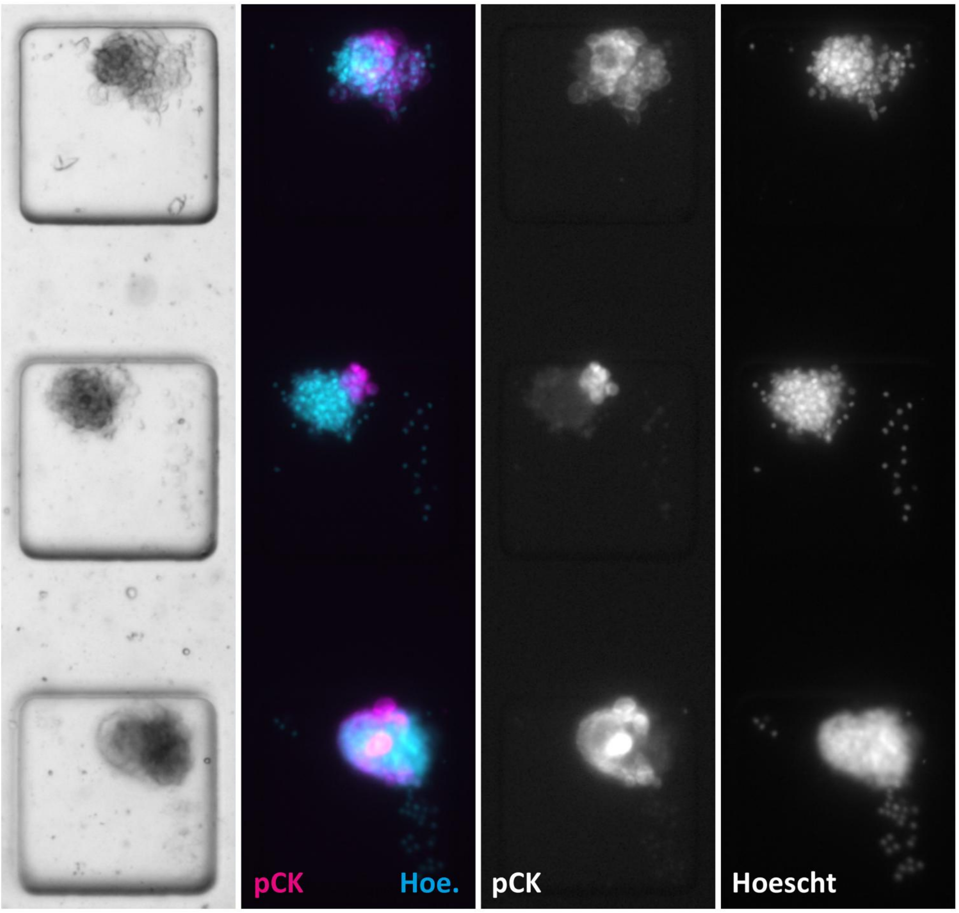

Supplementary Figure S1. Epithelia-stroma co-cultures. Representative images (on Day 4) showing cell organization within epithelia-stroma co-cultures (stroma seeded on Day 0, epithelia on Day 2), with the epithelial cells either wrapping around stromal aggregates (top and bottom wells) or forming a distinct cluster attached to the stromal cells (middle). The cultures were stained for pan-cytokeratin (pCK) and with the nucleic acid stain Hoescht 33324 (Hoe). The width of each microwell is 250 μm.
