# Supplementary material for: Functional, patient-derived 3D tri-culture models of the uterine wall in a microfluidic array
Source: Hum Reprod. 2024 Sep 15;39(11):2537–50. doi: 10.1093/humrep/deae214 (PMC11532614; doi:10.1093/humrep/deae214)
Supplement: deae214_Supplementary_Figure_S2 [file deae214_supplementary_figure_s2.pdf]

OXFORD

human reproduction

Human Reproduction, 2024, 1–1

https://doi.org/10.1093/humrep/deae214

Supplementary Data

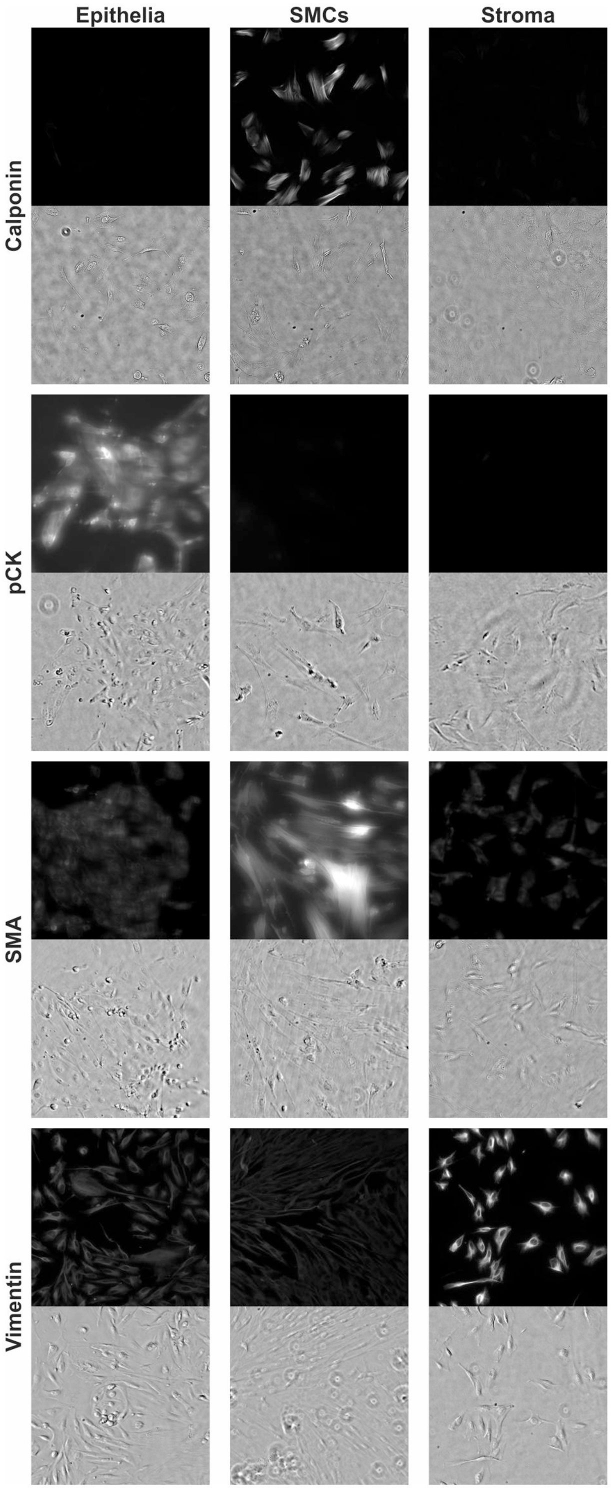

Supplementary Figure S2. Validation of immunocytochemistry (ICC) markers. Individual cell suspensions, obtained from either endometrial epithelial, endometrial stromal or myometrial fractions, were cultured in standard two dimensional (2D) conditions, fixed and stained with a single antibody to either calponin, pan-cytokeratin (pCK), alpha smooth muscle actin (SMA), or vimentin. All cultures for a given antibody were imaged under the same conditions and are shown with the same intensity range applied. Strong staining was obtained for only a single cell type for each antibody, validating the use of pCK as a marker for epithelia cells, vimentin for stromal cells and calponin/SMA for myocytes.
