# Supplementary material for: Functional, patient-derived 3D tri-culture models of the uterine wall in a microfluidic array
Source: Hum Reprod. 2024 Sep 15;39(11):2537–50. doi: 10.1093/humrep/deae214 (PMC11532614; doi:10.1093/humrep/deae214)
Supplement: deae214_Supplementary_Figure_S3 [file deae214_supplementary_figure_s3.pdf]

OXFORD
human reproduction
Human Reproduction, 2024, 1–1
https://doi.org/10.1093/humrep/deae214
Supplementary Data

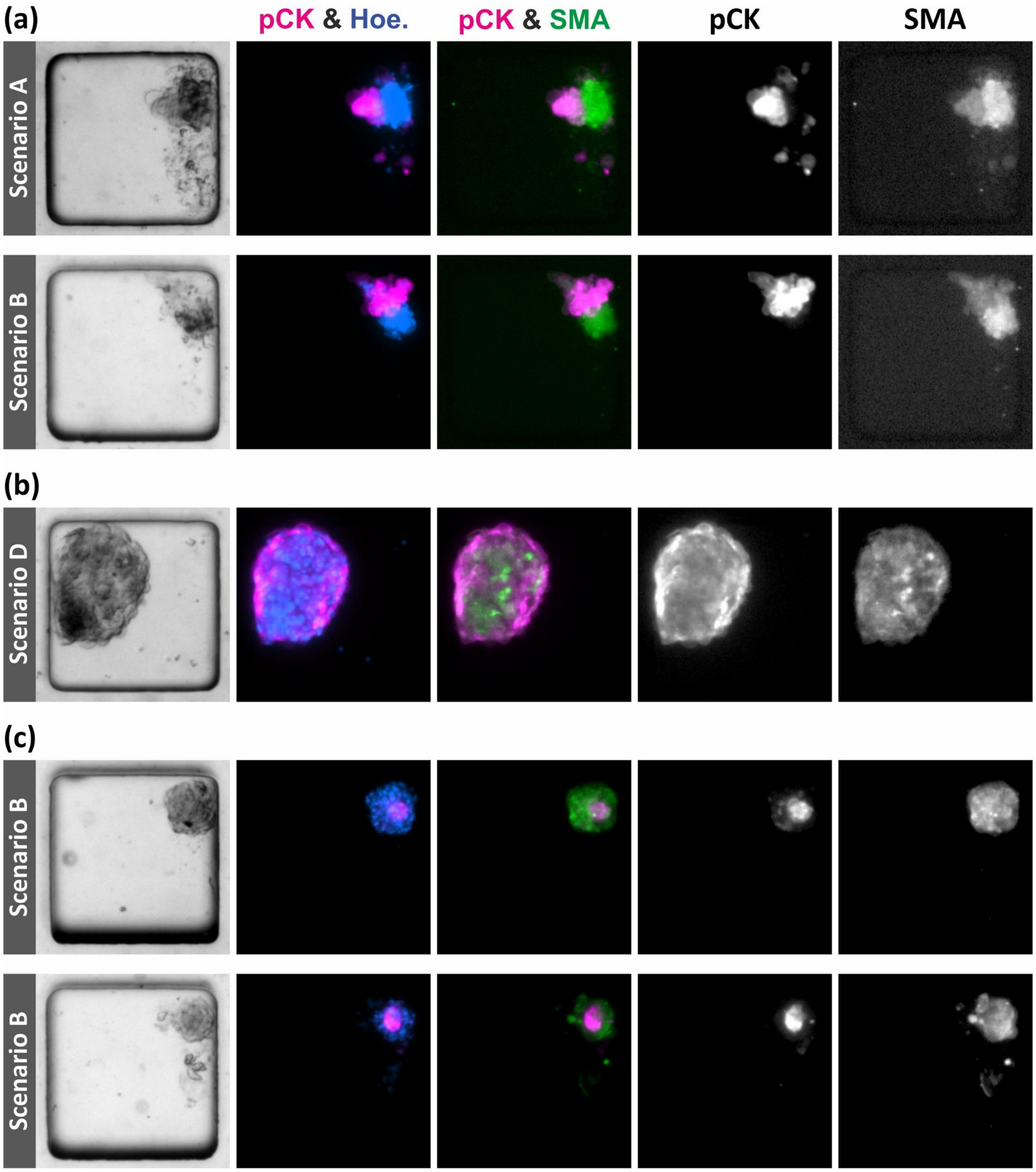

Supplementary Figure S3. Different cellular organizations within 3D uterine tri-cultures. Example images showing alternative cellular organization within the tri-cultures, showing examples of (**a**) segregation of epithelial clusters within a 3D aggregate; (**b**) larger 3D aggregates with the epithelial cells wrapping around a stromal-smooth muscle cell (SMC) core; and (**c**) rarer instances of an epithelial core surrounded by SMCs. The seeding scenarios used are noted for each set of images and refer to Fig. 2d. The cultures were stained for pan-cytokeratin (pCK), alpha smooth muscle actin (SMA) and with the nucleic acid stain Hoescht 33324 (Hoe). The width of each microwell is 250 μm.
