# Supplementary material for: Functional, patient-derived 3D tri-culture models of the uterine wall in a microfluidic array
Source: Hum Reprod. 2024 Sep 15;39(11):2537–50. doi: 10.1093/humrep/deae214 (PMC11532614; doi:10.1093/humrep/deae214)
Supplement: deae214_Supplementary_Figure_S4 [file deae214_supplementary_figure_s4.pdf]

OXFORD
human reproduction

Human Reproduction, 2024, 1–1
https://doi.org/10.1093/humrep/deae214
Supplementary Data

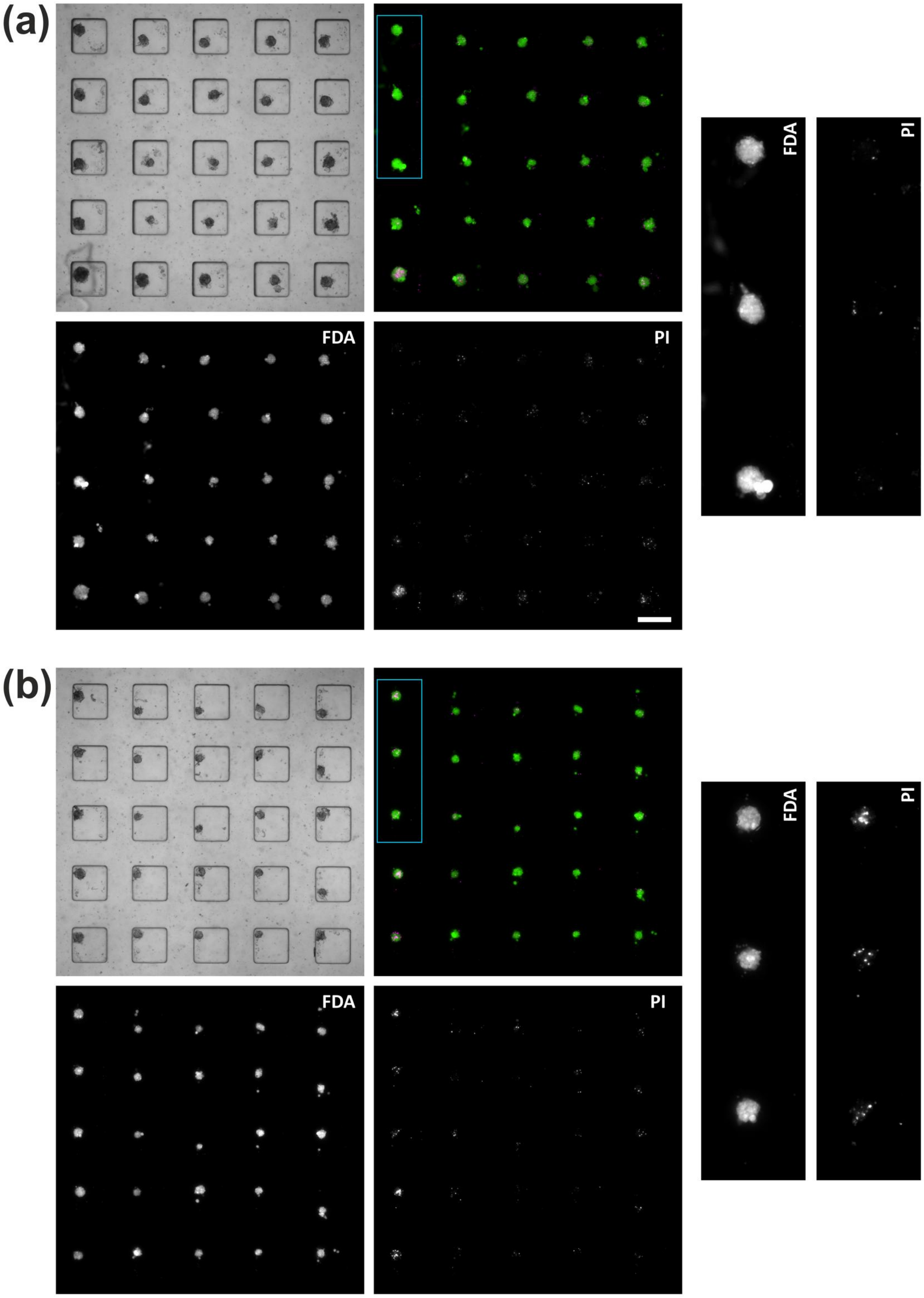

Supplementary Figure S4. Cell viability in 3D tri-cultures. Cultures were produced by sequentially seeding stromal cells followed by epithelia and lastly smooth mucle cells (SMCs) (Scenario D). Viable cells were stained with fluorescein diacetate (FDA, green in merged image) and dead cells with propidium iodide (PI, red in merged image). The blue boxes on the 5 × 5 array images mark the regions corresponding to the magnified images on the right-hand side. (**a**) Cultures assessed at Day 7. (**b**) Cultures assessed at Day 10.
