# Supplementary material for: Functional, patient-derived 3D tri-culture models of the uterine wall in a microfluidic array
Source: Hum Reprod. 2024 Sep 15;39(11):2537–50. doi: 10.1093/humrep/deae214 (PMC11532614; doi:10.1093/humrep/deae214)
Supplement: deae214_Supplementary_Figure_S5 [file deae214_supplementary_figure_s5.pdf]

OXFORD
human reproduction
Human Reproduction, 2024, 1–1
https://doi.org/10.1093/humrep/deae214
Supplementary Data

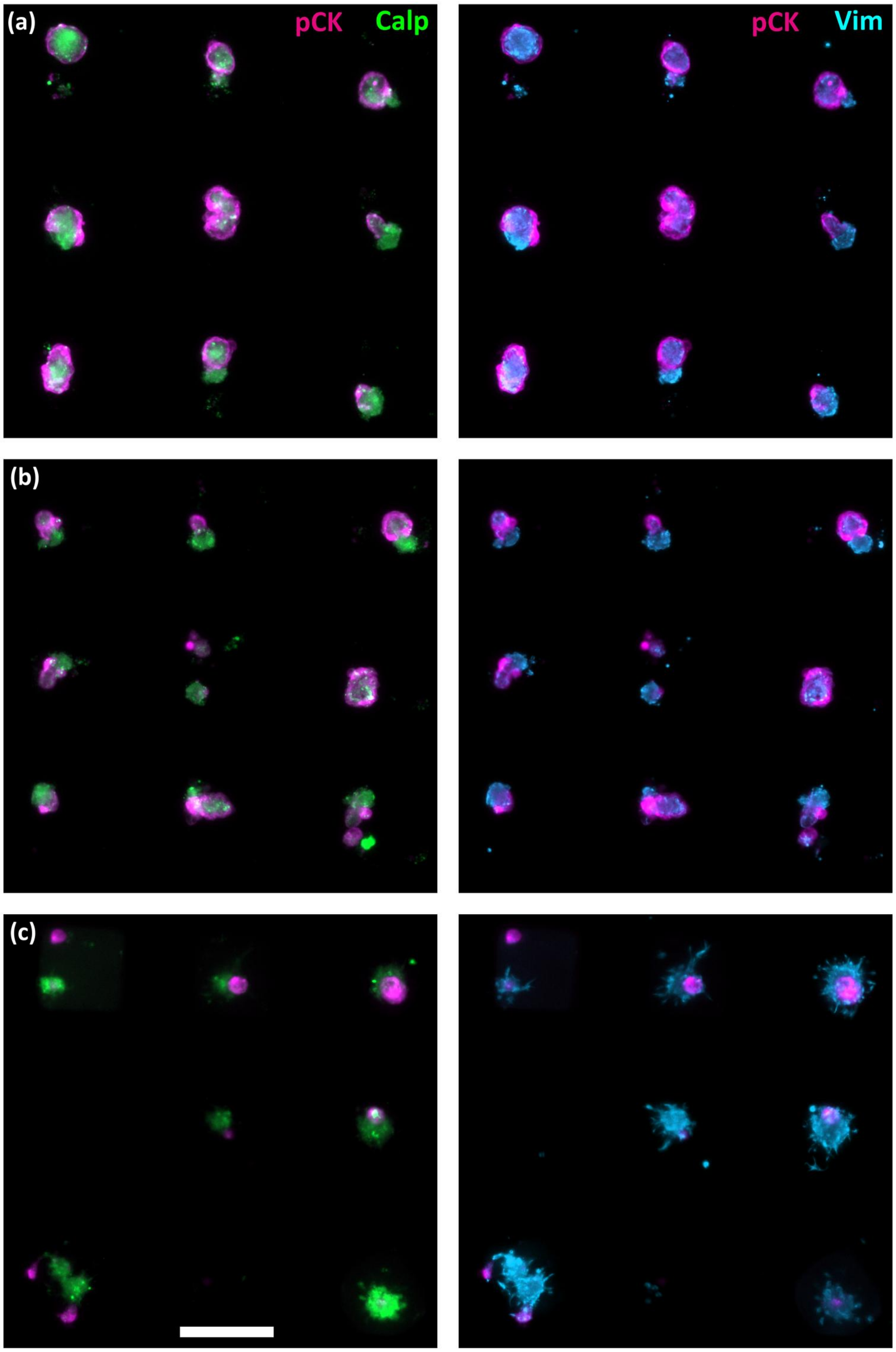

Supplementary Figure S5. Comparison of 3D culture architectures obtained with and without gel support. (**a**) A series of uterine tri-culture models within a microwell array, cultured in the presence of 5% Matrigel®. For clarity, staining for pan-cytokeratin (pCK, magenta) and calponin (Calp, green) are shown separately from pCK and vimentin (Vim, blue). (**b**) A corresponding series of uterine tri-culture models cultured in the absence of hydrogel. (**c**) A series of uterine tri-culture models, cultured in the presence of 100% Matrigel®, highlighting the pronounced outgrowth of stromal cells and the frequent segregation of the epithelium. All cultures (a–c) were fixed on Day 9 and followed the cell seeding timeline described in Fig. 3. The scale bar corresponds to 250 μm and applies to all images.
