# Supplementary material for: Functional, patient-derived 3D tri-culture models of the uterine wall in a microfluidic array
Source: Hum Reprod. 2024 Sep 15;39(11):2537–50. doi: 10.1093/humrep/deae214 (PMC11532614; doi:10.1093/humrep/deae214)
Supplement: deae214_Supplementary_Figure_S6 [file deae214_supplementary_figure_s6.pdf]

OXFORD
human reproduction
Human Reproduction, 2024, 1–1
https://doi.org/10.1093/humrep/deae214
Supplementary Data

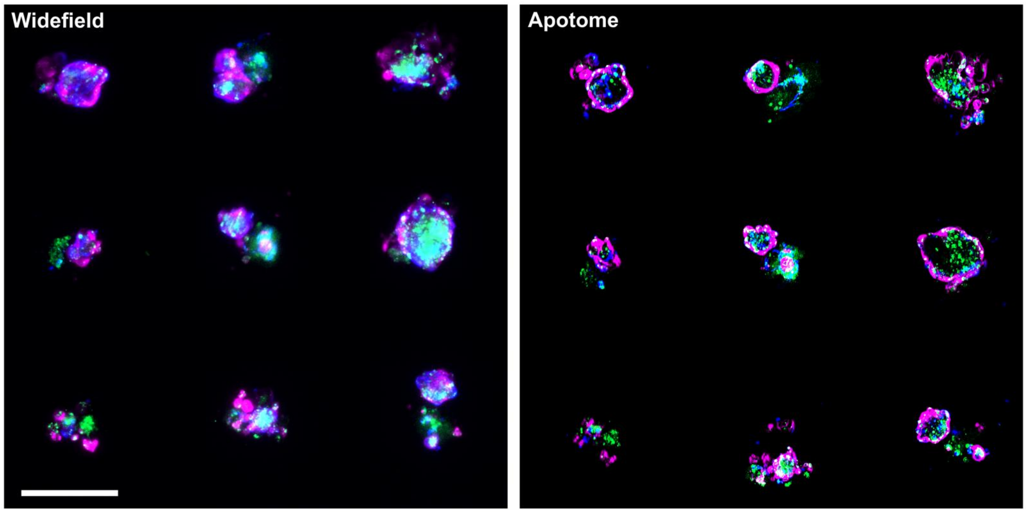

Supplementary Figure S6. Epithelial encapsulation of 3D tri-cultures within a microwell array. A 3 × 3 section of a microwell array, showing wide-field imaging on the left and optical sectioning by Apotome imaging on the right. Here, seven out of nine individual cultures show either complete or partial encapsulation (e.g. 'All', see main text for category definitions). Scale bar corresponds to 250 μm.
