# Supplementary material for: Functional, patient-derived 3D tri-culture models of the uterine wall in a microfluidic array
Source: Hum Reprod. 2024 Sep 15;39(11):2537–50. doi: 10.1093/humrep/deae214 (PMC11532614; doi:10.1093/humrep/deae214)
Supplement: deae214_Supplementary_Figure_S7 [file deae214_supplementary_figure_s7.pdf]

OXFORD
human reproduction

Human Reproduction, 2024, 1–1
https://doi.org/10.1093/humrep/deae214
Supplementary Data

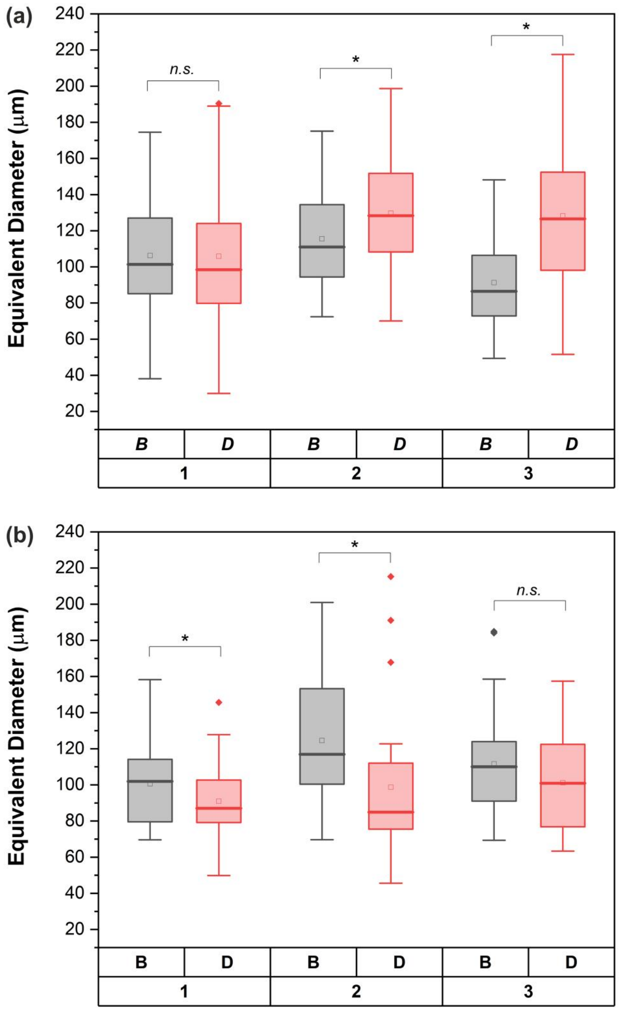

Supplementary Figure S7. Distribution of individual 3D culture sizes. Equivalent diameter values were calculated from area measurements obtained for each individual culture and are plotted for both hormone-stimulated cultures measured on Day 15 (**a**) and non-stimulated cultures measured on Day 9 (**b**), produced from three different patient samples and using two different seeding scenarios (B and D). Data, from two arrays per experimental condition, are displayed as box plots showing median (horizontal line) and mean (square) values, with * representing a significant difference between seeding scenarios B and D ($P < 0.05$, two-sample t-test).
