# Supplementary material for: Functional, patient-derived 3D tri-culture models of the uterine wall in a microfluidic array
Source: Hum Reprod. 2024 Sep 15;39(11):2537–50. doi: 10.1093/humrep/deae214 (PMC11532614; doi:10.1093/humrep/deae214)
Supplement: deae214_Supplementary_Figure_S8 [file deae214_supplementary_figure_s8.pdf]

OXFORD
human reproduction
Human Reproduction, 2024, 1–1
https://doi.org/10.1093/humrep/deae214
Supplementary Data

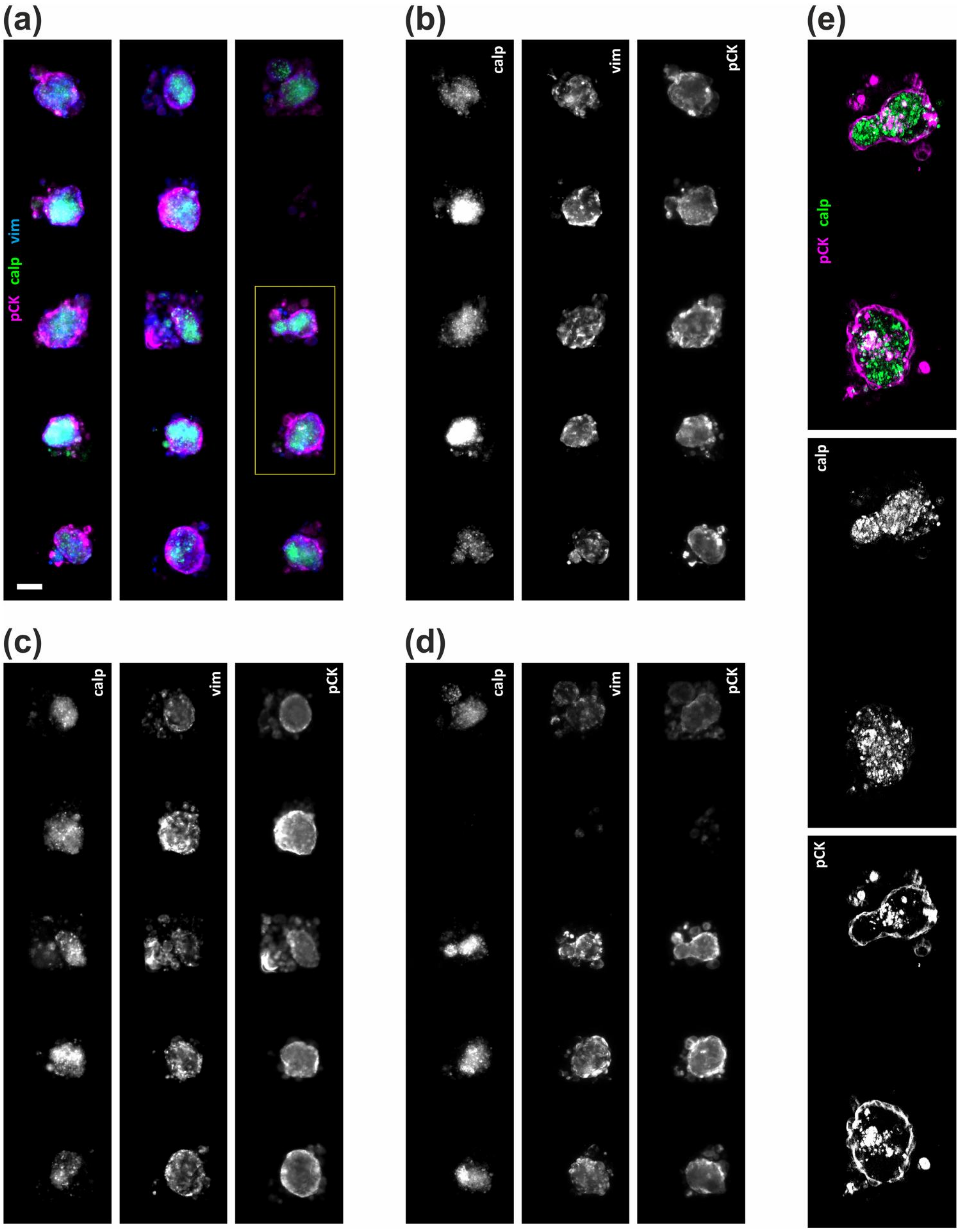

Supplementary Figure S8. Visual comparison of 3D tri-cultures produced from three different patient biopsies. Representative cultures from arrays seeded with cell fractions obtained from three different patients (the same three endometrial tissue donors as for Fig. 4), (**a**) showing a merged image for pCK (epithelia, magenta), calponin (SMC, green), and vimentin (stroma, blue) staining and (**b**–**d**) showing the individual fluorescent channels for each of these. (**e**) Shows higher magnification optical sectioning images of the boxed region in (a). Scale bar corresponds to 100 μm.
