# Supplementary material for: Functional, patient-derived 3D tri-culture models of the uterine wall in a microfluidic array
Source: Hum Reprod. 2024 Sep 15;39(11):2537–50. doi: 10.1093/humrep/deae214 (PMC11532614; doi:10.1093/humrep/deae214)
Supplement: deae214_Supplementary_Figure_S9 [file deae214_supplementary_figure_s9.pdf]

Human Reproduction, 2024, 1–1
https://doi.org/10.1093/humrep/deae214
Supplementary Data

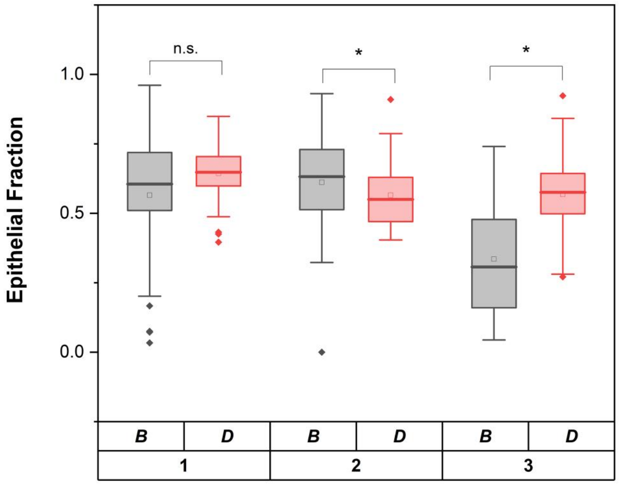

Supplementary Figure S9. The fraction of cells within an individual culture that were epithelial. By measuring the area of an individual culture that stained positive for the epithelial marker pCK (thresholding and processing as described in methods) and normalizing this to the total area defined by merging all fluorescent channels, a metric for the epithelial fraction of individual cultures was obtained. The data presented is for hormone-stimulated cultures, analysed at Day 15, with * representing a significant difference between seeding scenarios B and D ($P < 0.05$, two-sample *t*-test; n numbers as in Fig. 4c).
