# Supplementary material for: Functional, patient-derived 3D tri-culture models of the uterine wall in a microfluidic array
Source: Hum Reprod. 2024 Sep 15;39(11):2537–50. doi: 10.1093/humrep/deae214 (PMC11532614; doi:10.1093/humrep/deae214)
Supplement: deae214_Supplementary_Figure_S10 [file deae214_supplementary_figure_s10.pdf]

OXFORD
human reproduction
Human Reproduction, 2024, 1–1
https://doi.org/10.1093/humrep/deae214
Supplementary Data

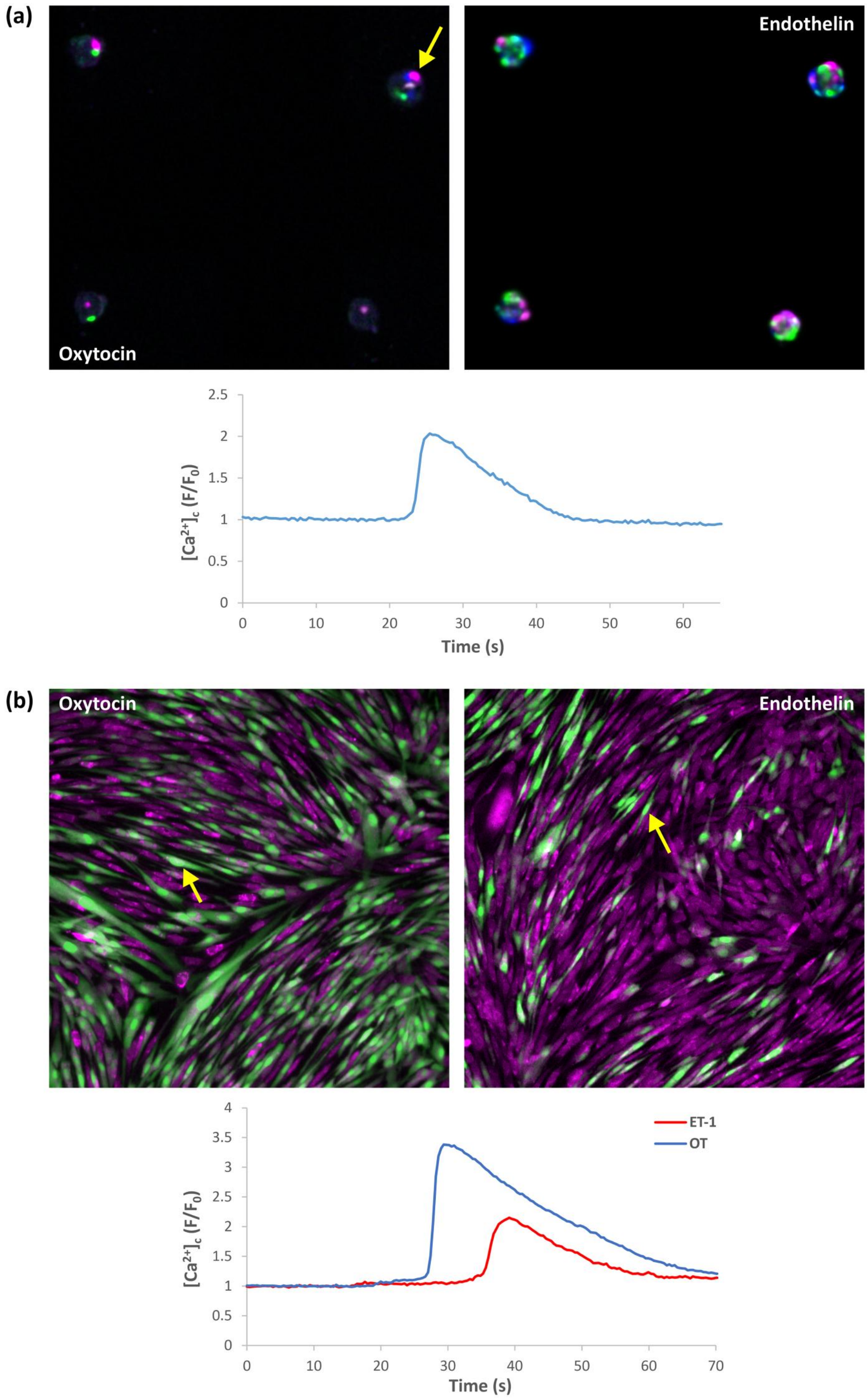

Supplementary Figure S10. The response of 2D and 3D smooth muscle cell (SMC) cultures to oxytocin (OT) stimulation. (**a**) The response of 3D SMC-only cultures to OT (left) and to ET-1 for the same cultures (right, ET-1 data from Fig. 5, shown side-by-side for comparison). Images show maps of responding cells, with the colour-coding corresponding to temporal variations in the response of individual cells (magenta showing cells that responded in the first 20 s period, green in the second 20 s period, blue in the third), whilst the representative trace below shows the response of the cell (magenta) highlighted by the yellow arrow. (**b**) The response of 2D SMC cultures to OT (left) and ET-1 (right), with images showing all viable cells (magenta) overlaid with all responding cells (green, cells responding within the first 60 s). The trace below shows representative temporal responses of the cells indicated by the yellow arrows.
