# Supplementary material for: Functional, patient-derived 3D tri-culture models of the uterine wall in a microfluidic array
Source: Hum Reprod. 2024 Sep 15;39(11):2537–50. doi: 10.1093/humrep/deae214 (PMC11532614; doi:10.1093/humrep/deae214)
Supplement: deae214_Supplementary_Table_S1 [file deae214_supplementary_table_s1.pdf]

Human Reproduction, 2024, 1–1
https://doi.org/10.1093/humrep/deae214
Supplementary Data

Supplementary Table S1. Participant demographics.

Donor	Menstrual phase	Experiments	Age	BMI	Parity	Type of caesarean	Gestation at delivery
Myometrial cells							
1	NA	Sequential seeding strategies, calcium imaging, ELISA	30	21.2	2	Elective non-labouring	39 + 2
2	NA	Sequential seeding strategies, calcium imaging, ELISA	40	35.0	3	Elective non-labouring	38 + 1
3	NA	Sequential seeding strategies, calcium imaging ELISA	29	24.1	4	Elective non-labouring	38 + 2
4	NA	Comparison of seeding scenarios B and D, 3D imaging	33	36.0	1	Elective non-labouring	39 + 4
Endometrial cells							
1	Secretory	Sequential seeding strategies, calcium imaging	28	37.5	2	NA	NA
2	Secretory	Sequential seeding strategies, calcium imaging	43	24.2	4	NA	NA
3	Late secretory	ELISA	32	21.9	3	NA	NA
4	Early secretory	ELISA	32	28.0	0	NA	NA
5	Mid-secretory	ELISA	45	29.5	2	NA	NA
6	Proliferative	ELISA	26	25.3	0	NA	NA
7	Secretory	Comparison of seeding scenarios B and D, 3D imaging	41	23.6	0	NA	NA
8	Secretory	Comparison of seeding scenarios B and D, 3D imaging	42	23.5	5	NA	NA
9	Secretory	Comparison of seeding scenarios B and D, 3D imaging	38	20.5	5	NA	NA

© The Author(s) 2024. Published by Oxford University Press on behalf of European Society of Human Reproduction and Embryology.
This is an Open Access article distributed under the terms of the Creative Commons Attribution License (https://creativecommons.org/licenses/by/4.0/), which permits unrestricted reuse, distribution, and reproduction in any medium, provided the original work is properly cited.
Human Reproduction, 2024, 00, 1–26
https://doi.org/10.1093/humrep/deae214
Original Article
